# Inhibition of the interaction between microglial adenosine 2A receptor and NLRP3 inflammasome attenuates neuroinflammation posttraumatic brain injury

**DOI:** 10.1111/cns.14408

**Published:** 2023-08-11

**Authors:** Hao Du, Chang‐Hong Li, Ruo‐Bing Gao, Yan Tan, Bo Wang, Yan Peng, Nan Yang, Ya‐Lei Ning, Ping Li, Yan Zhao, Yuan‐Guo Zhou

**Affiliations:** ^1^ Department of Army Occupational Disease, State Key Laboratory of Trauma and Chemical Poisoning, Research Institute of Surgery and Daping Hospital Army Medical University Chongqing China; ^2^ The General Hospital of Tibet Military Command Tibet China; ^3^ Department of Pathophysiology, College of High‐Altitude Military Medicine Army Medical University Chongqing China; ^4^ Institute of Brain and Intelligence Army Medical University Chongqing China

**Keywords:** adenosine 2A receptor, microglia, neuroinflammation, NLRP3 inflammasome, traumatic brain injury

## Abstract

**Aims:**

Adenosine 2A receptor (A_2A_R) is widely expressed in the brain and plays important roles in neuroinflammation, and the nucleotide‐binding oligomerization domain, leucine‐rich repeat, and pyrin domain‐containing protein 3 (NLRP3) inflammasome is a crucial component of the innate immune system while the regulation of A_2A_R on it in the central nervous system (CNS) has not been clarified.

**Methods:**

The effects of microglial A_2A_R on NLRP3 inflammasome assembly and activation were investigated in wild‐type, A_2A_R‐ or NLRP3‐knockout primary microglia with pharmacological treatment. Microglial A_2A_R or NLRP3 conditional knockout mice were used to interrogate the effects of this regulation on neuroinflammation posttraumatic brain injury (TBI).

**Results:**

We found that A_2A_R directly interacted with NLRP3 and facilitated NLRP3 inflammasome assembly and activation in primary microglia while having no effects on mRNA levels of inflammasome components. Inhibition of the interaction via A_2A_R agonist or knockout attenuated inflammasome assembly and activation in vitro. In the TBI model, microglial A_2A_R and NLRP3 were co‐expressed at high levels in microglia next to the peri‐injured cortex, and abrogating of this interaction by microglial NLRP3 or A_2A_R conditional knockout attenuated the neurological deficits and neuropathology post‐TBI via reducing the NLRP3 inflammasome activation.

**Conclusion:**

Our results demonstrated that inhibition of the interaction between A_2A_R and NLRP3 in microglia could mitigate the NLRP3 inflammasome assembly and activation and ameliorate the neuroinflammation post‐TBI. It provides new insights into the effects of A_2A_R on neuroinflammation regulation post‐TBI and offers a potential target for the treatment of NLRP3 inflammasome‐related CNS diseases.

## Graphical abstract

Microglial A_2A_R could interact with NLRP3 and regulate the NLRP3 inflammasome activation. Abrogating the interaction by conditional A_2A_R or NLRP3 knockout inhibits NLRP3 inflammasome activation in mice with posttraumatic brain injury.

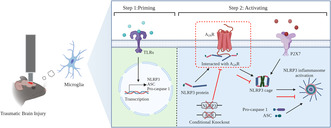

## 1 INTRODUCTION

Neuroinflammation plays a crucial role in many CNS diseases including TBI, Parkinson's disease (PD), and Alzheimer's disease (AD).^1^, ^2^, ^3^ Microglia are resident immune cells of the CNS and their overactivation is considered to be the main contributor to many neuroinflammation‐related diseases.^4^ Therefore, the regulation of neuroinflammation caused by microglia overactivation would be beneficial to diverse CNS diseases.

NLRP3 is mainly expressed in microglia in the CNS and is considered an important contributor to neuroinflammation in many diseases.^5^ The NLRP3 inflammasome composed NLRP3, apoptosis‐associated speck‐like protein (ASC), and pro‐caspase 1,^6^ and the inflammasome activation includes two steps: first, priming signals increase the expression of NLRP3 inflammasome components including NLRP3, ASC, pro‐caspase 1, and downstream effectors such as gasderminD (GSDMD), interleukin‐1β (IL‐1β), and interleukin‐18; next, activating signals result in the assembly and full activation of the NLRP3 inflammasome, following by the release of caspase 1 p20, N terminal part of GSDMD (N‐GSDMD), and matured interleukins.^7^ Many studies focused on the inflammasome assembly process to regulate its activation, and some NLRP3 inhibitors target the interactions between NLRP3 inflammasome components, such as MCC950, Oridonin, and Tranilast.^8^ It was also reported that the endogenous NIMA‐related kinase 7 (NEK7) could regulate NLRP3 inflammasome oligomerization and activation.^9^ Moreover, A_2A_R was reported to regulate the sustained NLRP3 inflammasome activation via cAMP/PKA/CREB/HIF‐1α pathway in macrophages.^10^ But the effects of A_2A_R on the NLRP3 inflammasome activation in the CNS remain to be elucidated.

A_2A_R is widely expressed in diverse cell types in the brain including microglia and it is involved in the regulation of neuroinflammation in many CNS diseases.^11^ A_2A_R activation exerts anti‐inflammatory effects on many types of diseases, while increasing glutamate concentrations, which commonly occurs in CNS diseases, could switch the anti‐inflammatory effect to pro‐inflammatory effect as we reported before.^12^, ^13^ Therefore, A_2A_R agonists were used for the treatment of peripheral system diseases while A_2A_R antagonist exerted neuroprotective effects in the CNS diseases.^14^ It has been reported that A_2A_R participated in regulating the NLRP3 inflammasome in macrophages and endothelial cells,^15^, ^16^ which contributed to better outcomes of bronchopulmonary dysplasia and stroke, and A_2A_R blockade could inhibit NLRP3 inflammasome activation and promote M2 like microglia polarization in the model of hypoxic–ischemic white‐matter damage.^17^ However, the regulatory effects of microglial A_2A_R on the NLRP3 inflammasome in the CNS and underlying mechanisms still need to be uncovered because of the opposite effects of A_2A_R activation in the peripheral and central nervous systems.

Hence, we investigated the underlying mechanisms of A_2A_R to regulate NLRP3 inflammasome in primary microglia and further explored the effects of this regulation on neuroinflammation in a TBI animal model. Our findings would provide new insights into the underlying mechanisms by which A_2A_R regulates NLRP3 inflammasome in microglia and contribute to the development of A_2A_R‐targeting drugs for NLRP3 inflammasome‐related CNS diseases.

## 2 MATERIALS AND METHODS

### 2.1 Animals

Adult male C57/BL6J mice (10–12 weeks) were used in animal studies. A_2A_R‐KO mice were obtained from Professor Jiang‐Fan Chen.^18^ NLRP3‐KO mice were obtained from Professor Lei Li (purchased from the Jackson Laboratory, stock number: 021302). A_2A_R^flox/flox^ mice were purchased from Shanghai Model Organisms Company (stock number: NM‐CKO‐210054). NLRP3^flox/flox^ mice were generated using the CRISPR/Cas9 technique by GemPharmaTech Company. CX3CR1^CRE/ERT2^ mice (CX3CR1^CRE^) were obtained from Professor Xiao‐Tang Fan (purchased from the Jackson Laboratory, stock number: 021160). The CX3CR1^CRE^ mice express the Cre‐recombinase under the control of CX3CR1 promoter after tamoxifen induction, which is mainly showed in microglia in the CNS, and the CX3CR1^CRE^ mice also expressed EYFP under the control of CX3CR1 promoter that provided a valuable marker for microglia. Microglial A_2A_R or NLRP3 conditional knockout mice (termed as A_2A_R^CX3CR1^ and NLRP3^CX3CR1^ mice, respectively) were generated by mating floxed mice with CX3CR1^CRE^ mice. The primers used for mouse genotyping are listed in Table S1 and representative results of agarose gel electrophoresis for genotyping are shown in Figure S1. Conditional activation of Cre‐recombinase was induced by a 5‐day consecutive intraperitoneal injection (75 mg/kg) of tamoxifen (Sigma, 20 mg/mL in corn oil).

After 1 month, brains from tamoxifen‐induced mice were used for magnetic‐activated cell sorting (MACS) (Miltenyi Biotec) to sort primary microglia, and then sorted cells were detected using western blots (WB) to verify the efficiency of microglial A_2A_R or NLRP3 knockout (Figure S2). Mice were housed in the animal center of Daping Hospital, Army Medical University (Certificate SCXK [Yu] 2002–0002, Chongqing, China). All animal experiments were approved by the Laboratory Animal Welfare and Ethics Committee of the Army Medical University (AMUWEC20191822) and complied with the ARRIVE guidelines and the NIH Guide for the Care and Use of Laboratory Animals.

### 2.2 Primary microglia sorting, culture and pharmacological treatment

P12‐15 mice were used for primary microglia sorting to obtain mature cells as recommended.^19^ Primary microglia were sorted with the MACS method combined with Percoll as reported before.^19^, ^20^ Homozygote A_2A_R or NLRP3 knockout mice and their wild‐type littermates were used to sort primary A_2A_R or NLRP3 knockout microglia and the control microglia. Briefly, the mice were anesthetized and the brain was dissected. Mouse brains were digested with trypsin and filtrated with a 75 μm cell strainer. Then, Percoll (Amersham, cat. no. 17089102) was used to remove myelin.^20^ Cell pellets were resuspended and incubated with anti‐CD11b microbeads for 15 min at 4°C (1:10, Miltenyi Biotec). Finally, the microglia were sorted by an LS column placed in a matched magnet. The purity of sorted microglia was identified using flow cytometry (using anti‐CD11b APC antibody, 1:100, Miltenyi Biotec).

The sorted microglia were resuspended in DMEM/F12 medium with 10% heat‐inactivated FBS (Gibco) and 20 ng/mL recombinant M‐CSF mouse protein (Beyotime) which could promote microglia ramification as reported.^21^ Then, the microglia were maintained in a humidified incubator at 37°C with 5% CO_2_.

One day before cell stimulation, the culture medium was replaced with medium without M‐CSF. The culture dishes were randomly separated into several groups as indicated in the figure legend to perform the different treatment. A_2A_R agonist CGS21680 (CGS) (TOCRIS, cat. no.1063) was dissolved in dimethyl sulfoxide (DMSO) at 50 mM and then diluted to 100 nM with DMEM/F12. Lipopolysaccharide (LPS, Sigma) and ATP (Sigma) were dissolved in the DMEM/F12 medium directly. For the CGS + LPS + ATP group, microglia were pretreated with 100 nM CGS for 30 min. Next, cells were treated with LPS at a final concentration of 1 μg/mL for 3 h followed by a 20‐min incubation with 1 mM ATP to fully activate NLRP3 inflammasome. The control and LPS + ATP groups were treated for 30 min with the same volume and concentration of DMEM/F12 containing DMSO as that in the CGS + LPS + ATP group.

### 2.3 Cell viability assay

Cell viability was detected with a cell counting kit‐8 assay (CCK‐8, Beyotime) according to the manufacturer's instructions. The absorbance at 450 nm was measured using a microplate reader, and the ratio to the control group was calculated.

### 2.4 Detection of LDH release

LDH release was measured with an LDH detection kit (Solarbio) according to the manufacturer's instructions. LDH activity was reported as the ratio of LDH released into the supernatant to the maximal LDH release (cells were lysed and mixed with the supernatant).^22^

### 2.5 Enzyme‐linked immunosorbent assay (ELISA)

Mouse IL‐1β (R&D, Valukine VAL601), IL‐18 (Sino Biological, KIT50073), and caspase 1 (Adipogen, AG‐45B‐0002) ELISA kits were used to detect IL‐1β, IL‐18, and caspase 1 p20 released in the supernatant under the manufacturer's instructions.^23^

### 2.6 Detection of caspase 1 activity

Caspase 1 activity was detected using a caspase 1 activity detection kit (Beyotime) according to the manufacturer's instructions.^24^ The absorbance at 405 nm was measured using a microplate reader which indicated the concentration of active caspase 1 in the sample. The ratio to the control group was calculated.

### 2.7 Western blots (WB)

WB were conducted as reported before.^25^ Briefly, proteins were electrophoresed and separated on 10% stain‐free SDS PAGE gels (Bio‐Rad) followed by transfer to PVDF membranes. Total protein was visualized with stain‐free technology of Bio‐Rad using the ChemiDoc imaging system (Bio‐Rad). Then, the membranes were blocked and incubated with specific primary antibodies overnight at 4°C. The antibodies used in this study are listed in Table S2. Five washes were applied after the incubation and then a horseradish peroxidase‐conjugated secondary antibody was incubated with the membranes at room temperature for 1 h. Finally, membranes were visualized using the ChemiDoc image system. Total protein was used as the loading control as it has been reported to be a more reliable control than housekeeping genes.^26^ All data were analyzed using Image Lab software (Bio‐Rad).

### 2.8 Quantitative PCR

Quantitative PCR was performed as reported previously.^27^ Briefly, total RNA was extracted with an RNA isolation kit (Promega). The RNA purity and concentration were detected using a NanoDrop One UV spectrophotometer followed by the adjusting of RNA concentration. Reverse transcription was conducted using a GoScript Reverse Transcription kit (Promega) according to the manufacturer's instructions. The cDNA was amplified using GoTaq qPCR mix (Promega) and the corresponding primers (Table S3).

### 2.9 Coimmunoprecipitation

Coimmunoprecipitation (Co‐IP) was carried out using Sera‐Mag Speed Beads Magnetic Protein A/G Particles (GE Life Sciences, 17152104011150) according to the manufacturer's protocol. Briefly, cells were lysed with IP lysis buffer (Pierce) containing a protease inhibitor cocktail. A portion of the protein lysates from each group were removed and mixed as the IgG group and another portion of all groups was denatured with SDS‐PAGE sample buffer as input and detected using WB. The Co‐IP samples were incubated with specific antibodies (anti‐NLRP3, 1:100, Adipogen, AG‐20B‐0014; anti‐A_2A_R, 1:100, Novus, NB300‐597) or normal IgG as a control overnight at 4°C with gentle shaking. The next day, the magnetic Protein A/G beads were washed with IP lysis buffer and then incubated with the sample/antibody mixture at room temperature for 1 h with mixing. Then, the beads were washed and collected with a magnetic stand. SDS‐PAGE sample buffer was added to the tube and heated for 10 min at 95°C. Finally, the beads were magnetically separated and the supernatant containing the target antigen was subjected to western blot detection, and conformation‐specific HRP‐conjugated secondary antibodies (CST) were used to avoid the interference of IgG chains. The ratio of the target protein to the control group was calculated.

### 2.10 TBI model

Previous studies showed that M1‐like microglia/macrophages increased from Day 3 and peaked at Days 5–7, and the number of microglia peaked at Day 7 during the acute stage post‐TBI.^28^, ^29^ Therefore, neurobehavioral tests were performed at 3‐ and 7‐d post‐TBI, and the pathological detection was performed at 7‐d post‐TBI. The controlled cortical impact method was used to produce moderate TBI models as described in our previous work.^30^ A_2A_R^CX3CR1^ or NLRP3^CX3CR1^ mice and their littermate CX3CR1^CRE^ mice were used in this study as an experimental or sham group. Briefly, mice were anesthetized with 2.5% tribromoethanol (250 mg/kg) and then subjected to a 5‐mm diameter craniotomy over the left parietal cortex. The center was placed between the bregma and the lambdoid suture. An aerodynamic impact device (PSI, USA) with a 3‐mm diameter metal tip was used to produce the controlled cortical impact (2 mm below the dura, 3.5 m/s impact speed). Sham animals underwent the same procedure as TBI mice except for the impact. The total number of mice used in each test is shown in Table S4. The exact number of biological replicates was also shown in figure legends. In general, 32 and 52 mice were used in the sham groups and TBI groups, respectively.

### 2.11 Microglia cell sorting with flow cytometry

To isolate microglia for WB analysis, TBI‐ or sham‐treated wild‐type mice were anesthetized, and the brain was dissected. Then the injured‐ or sham‐treated cortex was collected and digested using 0.25% trypsin. After being filtered with a 75 μm cell strainer, it was resuspended into 35% Percoll and centrifugated at 400 g to remove myelin as reported.^20^ Then cell pellets were resuspended in PBS and labeled with anti‐CD11b‐APC (Miltenyi, 1:50) and anti‐CD45‐FITC (BD, 1:100). CD11b and CD45 were used for distinguishing resident microglia (CD11b^+^, CD45^low^), activated microglia (CD11b^+^, CD45^intermediate^), and infiltrating macrophages (CD11b^+^, CD45^high^) as reported.^31^ Gates were established using antibody isotype controls and fluorescence minus‐one controls. Samples were acquired by Beckman MoFlo Astrios Cell Sorter and analyzed with Flowjo 10.0 software. Only CD11b^+^, CD45^low^, and CD11b^+^, CD45^intermediate^ cells were acquired, which indicated the microglia in the cortex specifically. Samples were then denatured with an SDS‐PAGE loading buffer and subjected to WB detection.

### 2.12 Immunofluorescence staining and image analysis

Immunofluorescence staining was performed as previously described.^25^ Briefly, 30‐μm‐thick coronal sections of fixed mice brains were prepared using a cryotome. Brain slices or cultured cells were washed with PBS and fixed with 4% PFA, followed by penetration with 0.3% Triton X‐100. Then, they were blocked using 10% goat serum. The primary antibody solution was added after blocking and incubated at 4°C overnight with gentle shaking. The next day, the samples were washed with PBS and incubated with the corresponding secondary antibody solution. DAPI (10 μg/mL, Sigma) was used for nuclear labeling. Finally, samples were mounted using an anti‐fade‐mounting medium after washing. The anti‐GFP antibody was used for labeling microglia as there was EYFP expressing under the control of the CX3CR1 promotor. Other antibodies and dilutions used in this article are shown in Table S2. All secondary antibodies were purchased from Abcam.

ASC speck‐positive (ASC speck^+^) cells were counted with ImageJ software as reported.^32^ All immunofluorescence data from brain slices were calculated with ImageJ software. Because of the lack of MAP2‐positive and NeuN‐positive cells (MAP2^+^, NeuN^+^) proximal to the injured area (Figure S3), images 300 μm from the injury site were acquired to investigate the effects of TBI on dendrites/axons and neurons. Three mouse brains and three slices containing injured sites from each brain were used to detect each target molecule of interest.

### 2.13 Neurobehavioral testing

A_2A_R^CX3CR1^ mice, NLRP3^CX3CR1^ mice, and their CX3CR1^CRE^ littermates were tested for neurofunctions before TBI to exclude the differences caused by conditional gene knockout. All mice scored 0 in the neurological severity score test and exhibited fewer than 5‐foot faults in the beam walk test. The open field and accelerating rotarod test results are shown in Figure S4, which indicated no significant differences among them. All animals were randomly selected and all tests were conducted by lab technicians who were blinded to the genotype and treatment of the experimental animals.

#### 2.13.1 Neurological severity score (NSS) test

Mice were scored for neurological severity using the method reported before.^33^ The scores range from 0 to 20, with 0 indicating normal function and 20 indicating the most severe injury.

#### 2.13.2 Open field test

Spontaneous locomotor activity and anxiety‐like behavior were assessed using the open field test as reported.^34^ The total distance traveled and time spent in the center area and in the perimeter were recorded during a 5‐min recording period.

#### 2.13.3 Beam walk test

The fine motor coordination of TBI mice was assessed by a beam walk test as described previously.^35^ Briefly, mice were placed on a 1‐cm wide wooden beam, and the number of foot faults of the right hindlimb was recorded over 50 steps. Mice were trained at 3 days before sham surgery or TBI.

#### 2.13.4 Accelerating rotarod test

Gross motor function and balance were assessed on the accelerating rotarod as reported previously.^35^ Mice were placed on the rod, which accelerated from 4 to 40 RPM within 300 s. The latency to fall from the rod (or cling to and rotate with the rod) was recorded. Mice were habituated to 4 RPM rotating at 3 days before sham surgery or TBI. Each mouse was performed in three trials, and the latency times were averaged.

### 2.14 Statistics

All data are presented as the means ± SEM and passed the Kolmogorov–Smirnov test for normality (*p* > 0.1). The behavioral data were analyzed using two‐way ANOVA followed by Tukey's multiple comparisons test. Other data were analyzed using the two‐tailed *t* test for comparison between two groups or one‐way ANOVA followed by Tukey's multiple comparisons test for three groups. All data were analyzed with GraphPad Prism 8 software. *p* value <0.05 was considered statistically significant.

## 3 RESULTS

### 3.1 A_2A_R agonist inhibits NLRP3 inflammasome activation‐induced inflammatory cytokine release and pyroptosis in primary microglia

**FIGURE 1:**
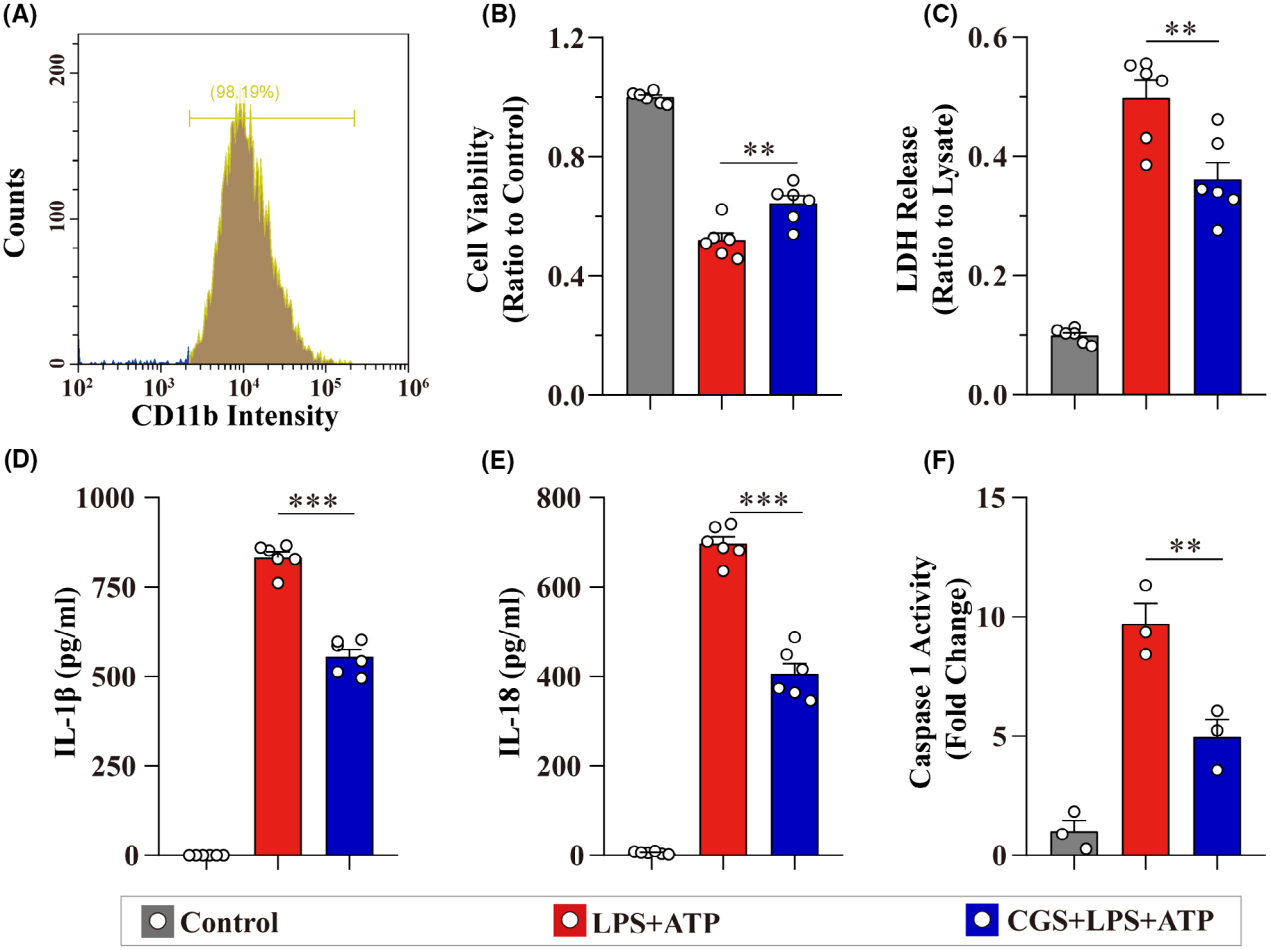
A_2A_R agonist inhibits NLRP3 inflammasome activation‐induced inflammatory cytokine release and pyroptosis in primary microglia. (A) The purity of sorted primary microglia was determined using flow cytometry. (B, C) Cell viability and LDH release from primary microglia under various treatments (*n* = 6). (D, E) The release of IL‐1β and IL‐18 were detected using ELISA (*n* = 6). (F) The caspase 1 activity in different groups (*n* = 3). Data are presented as the mean ± SEM and passed the Kolmogorov–Smirnov test for normality (*p* > 0.1), and then data were analyzed using one‐way ANOVA followed by Tukey's multiple comparisons test for significance. **p < 0.01 and ***p < 0.001.

Primary microglia sorted using MACS were verified with flow cytometry, and the purity was higher than 98% (Figure 1A). The cell death and LDH release induced by LPS + ATP were ameliorated by the A_2A_R agonist CGS (Figure 1B,C). IL‐1β and IL‐18 release were also reduced by CGS (Figure 1D,E). Moreover, the caspase 1 activity, which revealed the activation of NLRP3 inflammasome, was significantly inhibited by CGS (Figure 1F). These data demonstrated that A_2A_R agonist diminished NLRP3 inflammasome activation‐induced inflammatory cytokine release and pyroptosis in primary microglia.

### 3.2 A_2A_R agonist suppressed the cleavage of pro‐caspase 1 and full‐length GSDMD but did not alter mRNA levels of NLRP3 inflammasome components in microglia

**FIGURE 2:**
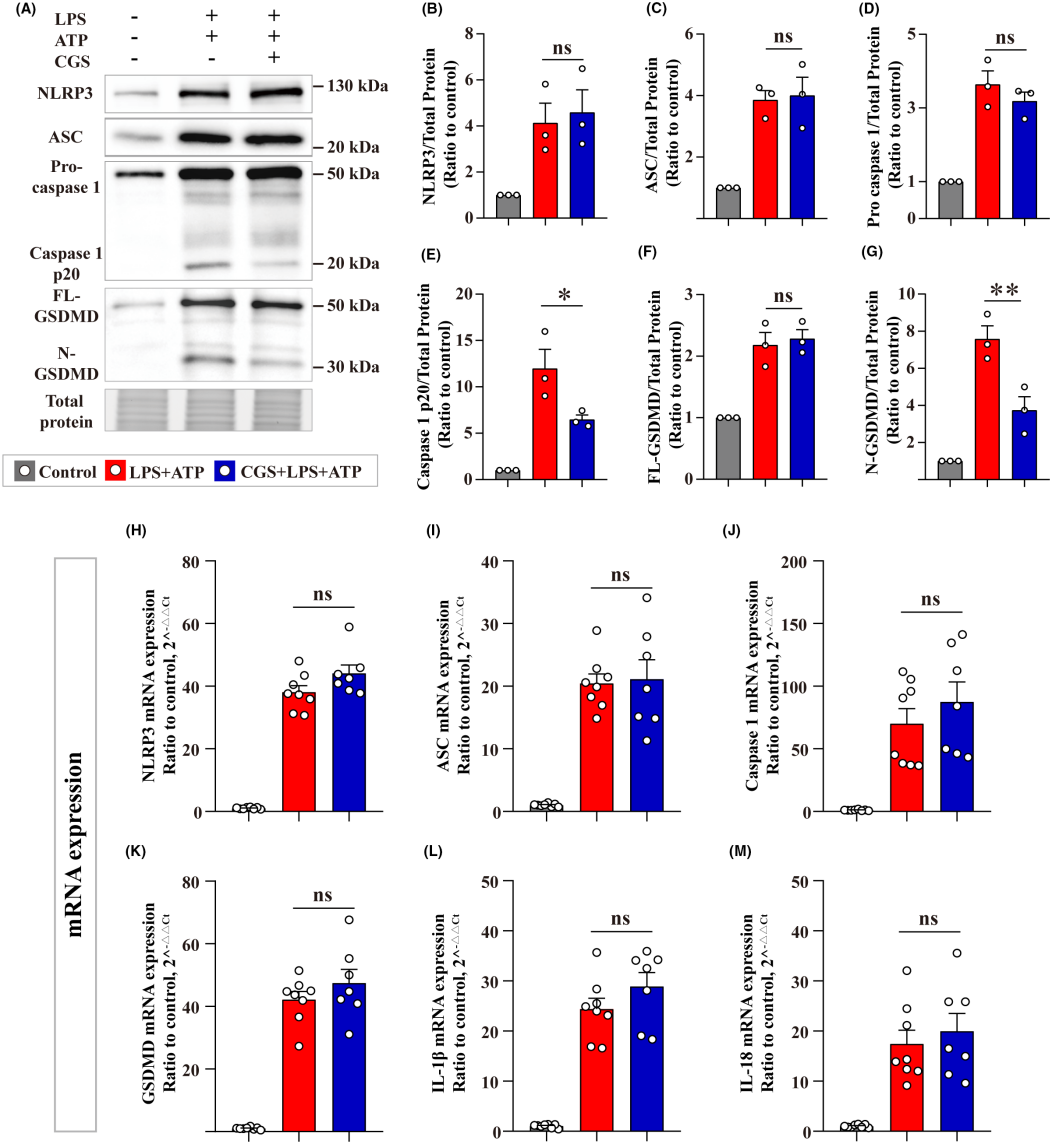
A_2A_R agonist suppressed pro‐caspase 1 and FL‐GSDMD cleavage without affecting mRNA levels of inflammasome components. (A) Representative images of immunoblots for NLRP3, ASC, pro‐caspase 1, caspase 1 p20, FL‐GSDMD, N‐GSDMD, and total protein image acquired with stain‐free technology of Bio‐Rad (a portion of full lane images were shown). (B–G) Quantitative analysis of the indicated proteins (*n* = 3). (H–M) The mRNA levels of NLRP3, ASC, caspase 1, GSDMD, IL‐1β, and IL‐18 were detected using qPCR (*n* = 7–8). Data are presented as the mean ± SEM and passed the Kolmogorov–Smirnov test for normality (*p* > 0.1), and then data were analyzed using one‐way ANOVA followed by Tukey's multiple comparisons test for significance. ns: *p* > 0.05, *p < 0.05, **p < 0.01.

Next, the effects of CGS on the expression and cleavage of NLRP3 inflammasome components were investigated in primary microglia. We found that CGS did not alter the protein levels of NLRP3 inflammasome‐related components, including NLRP3, ASC, pro‐caspase 1, and full‐length gasdermin D (FL‐GSDMD), while protein levels of caspase 1 p20 and N terminal part of GSDMD (N‐GSDMD) were reduced by CGS, indicating that the cleavage of pro‐caspase 1 and FL‐GSDMD was inhibited (Figure 2A–G). To further explore the effects of CGS on the expression of NLRP3 inflammasome components, the mRNA levels of NLRP3, ASC, caspase 1, GSDMD, IL‐1β, and IL‐18 were detected (Figure 2H–M). The results demonstrated that CGS had no effects on the mRNA levels of NLRP3 inflammasome‐related components.

### 3.3 Microglial A_2A_R interacted with NLRP3 and its inhibition diminished the NLRP3 inflammasome assembly

**FIGURE 3:**
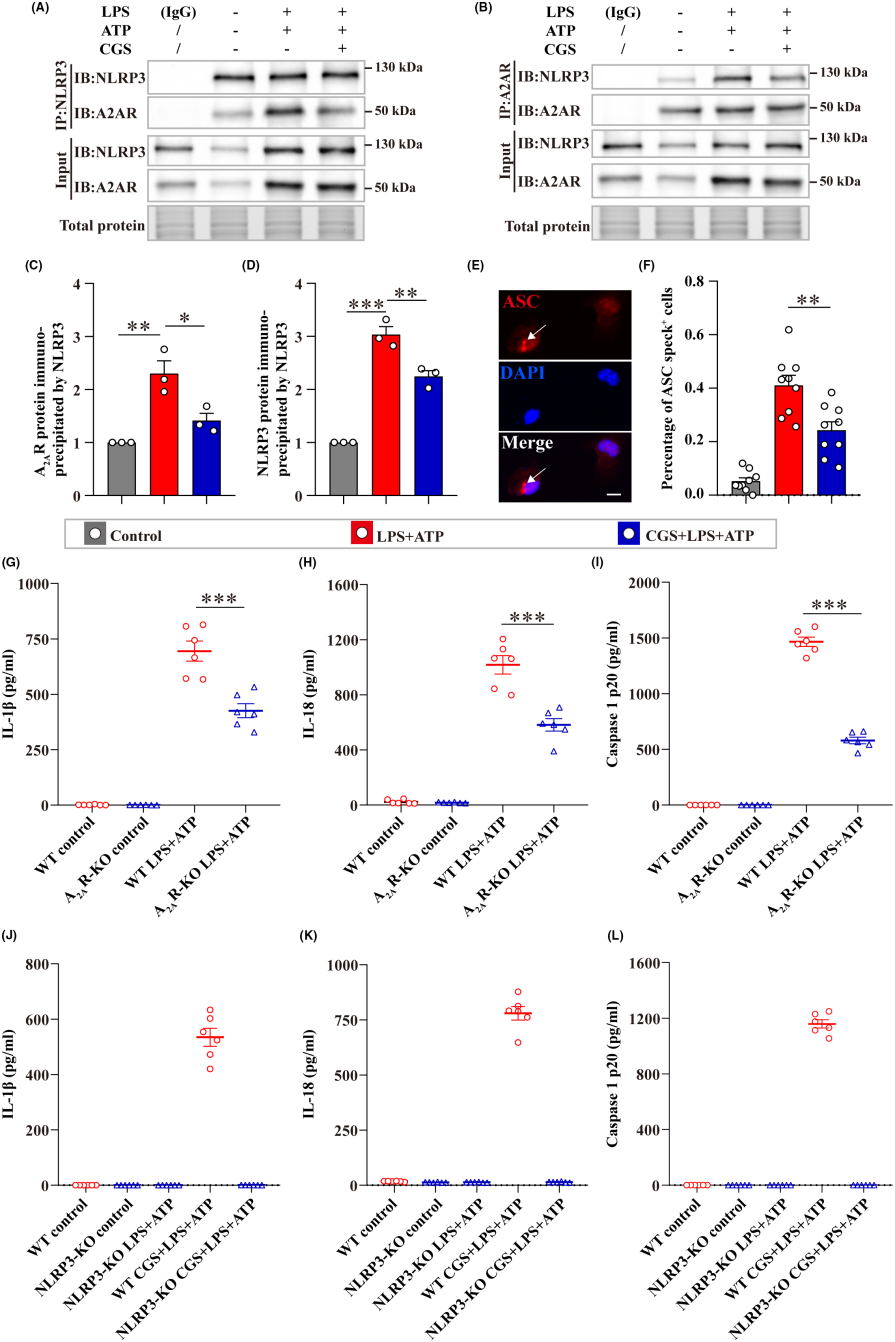
Microglial A_2A_R interacted with NLRP3 and its inhibition diminished the NLRP3 inflammasome assembly. (A, B) Representative images of A_2A_R and NLRP3 coimmunoprecipitated with the indicated antibodies and 10% input for each group detected using WB. Total protein blots were acquired with stain‐free technology of Bio‐Rad (a portion of full lane images were shown). The isotype control was set by adding normal IgG to the protein lysate and then treating it the same as other groups (*n* = 3). (C, D) Quantitative analysis of A_2A_R and NLRP3 protein levels coimmunoprecipitated with the indicated antibodies (*n* = 3). All data were normalized to the control group. (E) Representative images of immunofluorescence staining for the ASC speck^+^ cell (white arrow) and the ASC speck^−^ cell. Scale bar = 10 μm. (F) Quantitative analysis of ASC speck^+^ cells in different groups (*n* = 3, three randomly selected images for each sample). (G–L) The release of IL‐1β, IL‐18, and caspase 1 p20 from A_2A_R‐KO or NLRP3‐KO microglia, respectively (*n* = 6). Data are presented as the mean ± SEM and passed the Kolmogorov–Smirnov test for normality (*p* > 0.1), and then data were analyzed using one‐way ANOVA followed by Tukey's multiple comparisons test for significance. *p < 0.05, **p < 0.01, and ***p < 0.001.

As A_2A_R did not alter the mRNA levels of NLRP3 inflammasome components, the direct interaction between A_2A_R and NLRP3 was then investigated in primary microglia using Co‐IP (Figure 3A,B). The results revealed that LPS + ATP enhanced the interaction between A_2A_R and NLRP3 than the negative control, while CGS treatment reduced this interaction (Figure 3C,D). Then, the effects of CGS on the assembly of NLRP3 inflammasome was assessed by detecting ASC speck^+^ cells (Figure 3E,F). The data showed that CGS reduced LPS + ATP‐induced ASC oligomerization in primary microglia, which demonstrated that CGS treatment inhibited the NLRP3 inflammasome assembly (Figure 3F). Moreover, abrogating of this interaction by A_2A_R‐knockout (A_2A_R‐KO) or NLRP3‐knockout (NLRP3‐KO) also showed significant inhibition to the release of IL‐1β, IL‐18, and caspase 1 p20 after LPS + ATP stimulation (Figure 3G–L). These data demonstrated microglial A_2A_R could interact with NLRP3 and the inhibition of this interaction diminishes NLRP3 inflammasome assembly and downstream inflammatory effects.

### 3.4 The levels of NLRP3 and A_2A_R in microglia elevated at 7‐d post‐TBI

**FIGURE 4:**
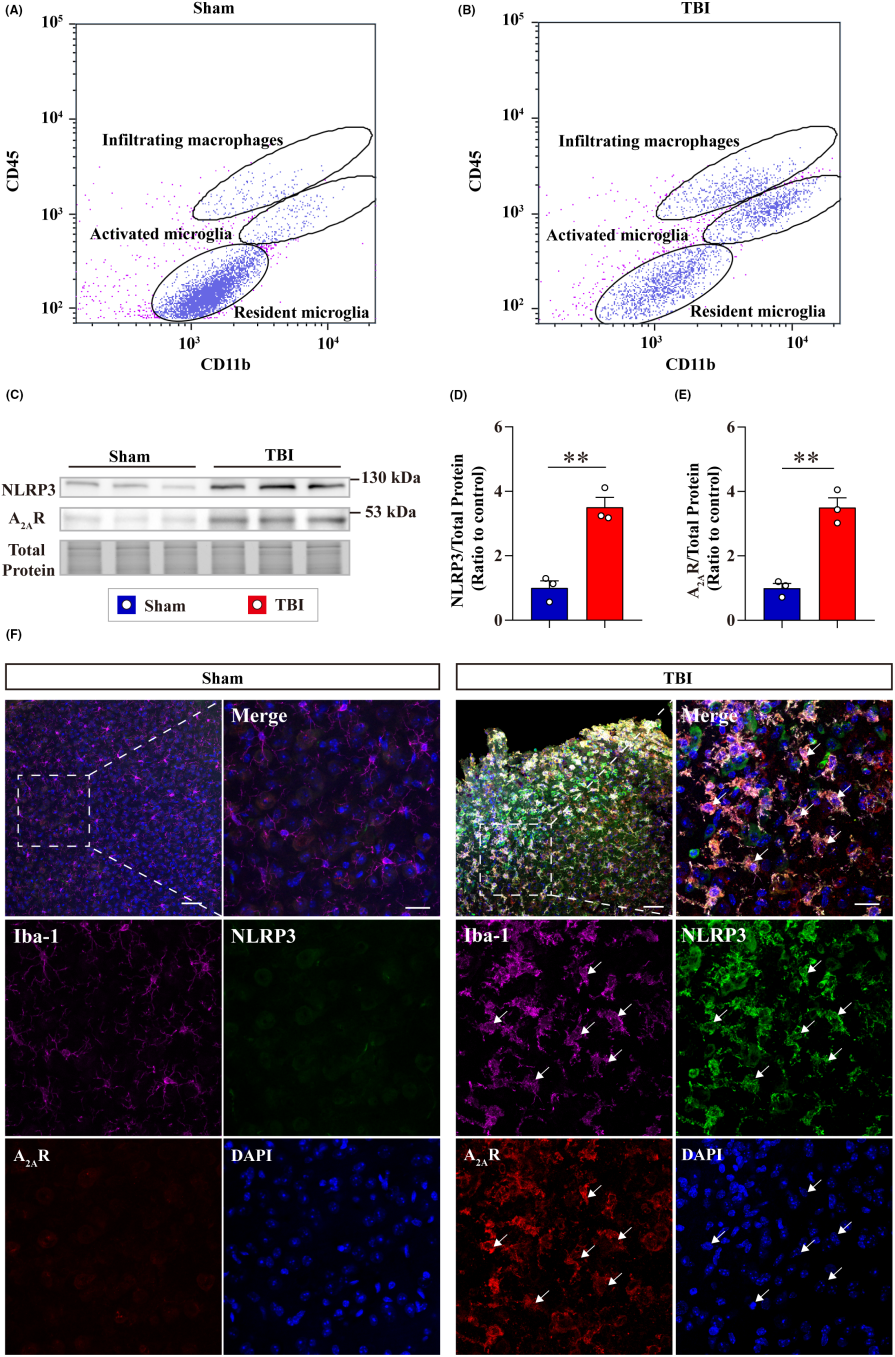
The levels of NLRP3 and A_2A_R in microglia elevated at 7‐d post‐TBI. (A, B) Representative flow cytometry density plots from the sham‐treated cortex and 7‐d post‐TBI cortex. Cells were labeled with CD11b and CD45. (C) Immunoblot images of NLRP3 and A_2A_R from flow cytometry sorted microglia in the sham and TBI cortex. (D, E) Quantitative analysis of NLRP3 and A_2A_R levels (*n* = 3, each sample indicated total microglia sorted from three mice brains). (F) Representative images of NLRP3, A_2A_R, and Iba‐1 immunofluorescence staining in the peri‐injured cortex at 7‐d post‐TBI and in the sham cortex (Iba‐1^+^, magenta; NLRP3^+^, green; A_2A_R, red; DAPI^+^, blue). scale bar = 100 μm for the top left image; scale bar = 20 μm for the top right image. The middle and bottom panels indicated the split channel images of the corresponding top right image. White arrows indicate the triple positive cells. Data are presented as the mean ± SEM and passed the Kolmogorov–Smirnov test for normality (*p* > 0.1), and then data were analyzed using the two‐tailed t test for significance. **p < 0.01.

Next, the regulatory effects of A_2A_R on NLRP3 inflammasome in neuroinflammation progression were investigated in a TBI model. The changes of A_2A_R and NLRP3 in microglia post‐TBI were examined first. Flow cytometry was performed to separate populations of microglia and infiltrating macrophages in the cortex at 7‐d post‐TBI. Cells from injured or sham‐treated cortex were labeled with CD11b and CD45 and only CD11b^+^, CD45^low^, and CD11b^+^, CD45^intermediate^ cells were collected, which indicated the resident and activated microglia, respectively (Figure 4A,B). The levels of NLRP3 and A_2A_R in the sorted microglia increased significantly at 7‐d post‐TBI as demonstrated by WB (Figure 4C–E), and immunofluorescence staining also showed that levels of A_2A_R and NLRP3 elevated at the peri‐injuried cortex and abundant of them were colocalized in Iba‐1^+^ microglia (Figure 4F). The increasing levels and colocalization of A_2A_R and NLRP3 implicate the significant roles of them in neuroinflammation post‐TBI.

### 3.5 Microglial NLRP3 conditional knockout attenuated neurological deficits and neuropathology post‐TBI

**FIGURE 5:**
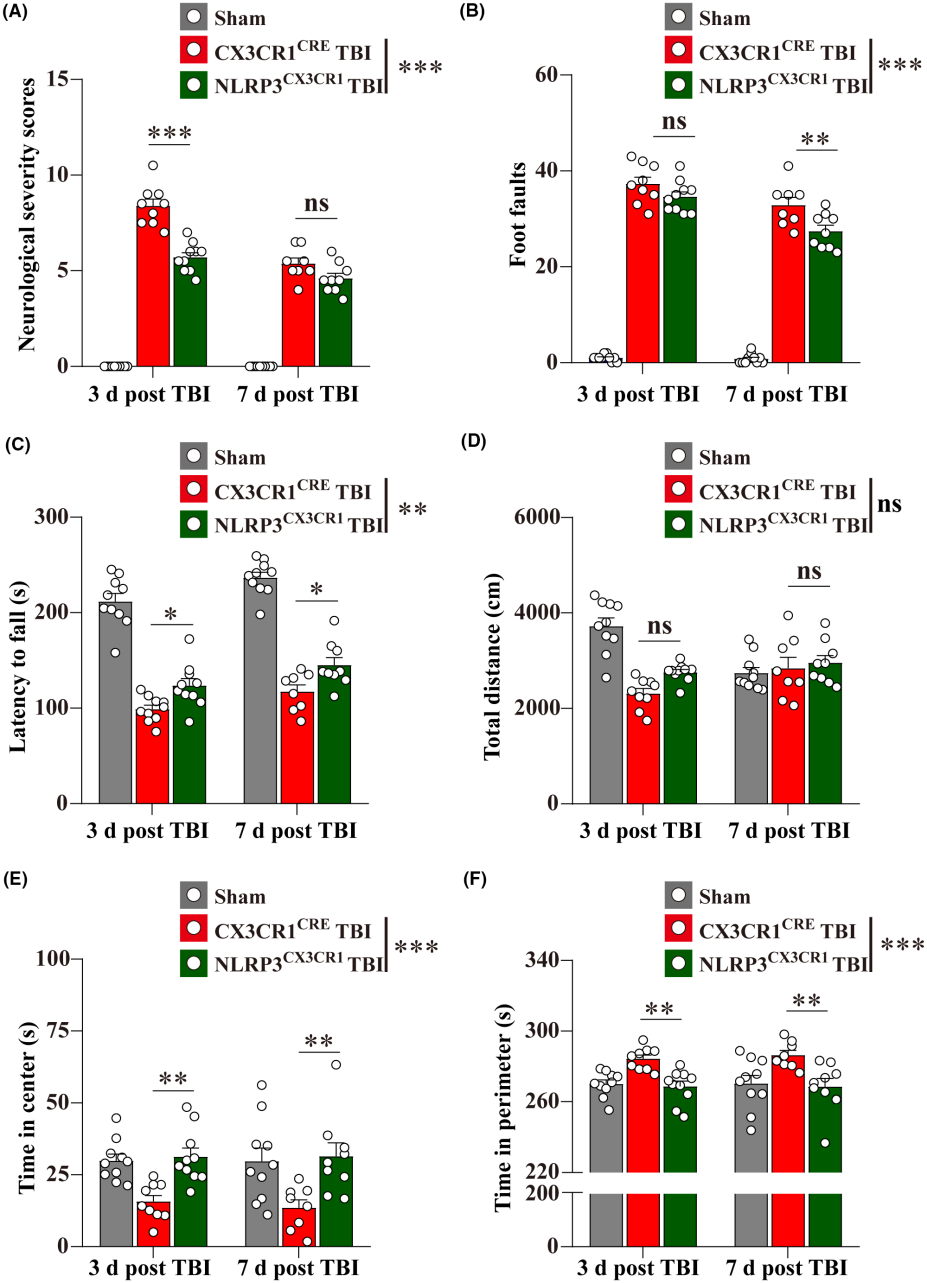
Microglial NLRP3 conditional knockout ameliorated neurological deficits at 3‐ and 7‐d post‐TBI. (A–C) The results of the NSS score, beam walk test, and the accelerating rotarod test (*n* = 9–10). (D–F) Total distance traveled (cm), time spent in center(s), and time spent in perimeter(s) were recorded in the open field test (*n* = 9–10). Data are presented as the mean ± SEM and passed the Kolmogorov–Smirnov test for normality (*p* > 0.1), and then data were analyzed using two‐way ANOVA followed by Tukey's multiple comparisons test for comparison at each time point. ns: *p* > 0.05, *p < 0.05, **p < 0.01, and ***p < 0.001.

Microglial NLRP3 conditional knockout mice (NLRP3^CX3CR1^ mice) and matched littermate control CX3CR1^CRE^ mice (used in the sham and CX3CR1^CRE^ TBI groups) were used for investigating the effects of abrogating the interaction between A_2A_R and NLRP3 on the neurological deficits and neuropathology post‐TBI. Before TBI, there were no significant differences between NLRP3^CX3CR1^ mice and their CX3CR1^CRE^ littermates in behavioral tests used in the article (Figure S4A–D). Then behavioral tests were performed to investigate the neurological deficits at 3‐ and 7‐d post‐TBI. The results demonstrated that the NLRP3^CX3CR1^ TBI group showed lower scores in the NSS test, fewer foot faults in the beam walk test, and longer latency to fall in the accelerating rotarod test than the CX3CR1^CRE^ TBI group (Figure 5A–C). In the open field test, there were no significant differences in the total distance between NLRP3^CX3CR1^ and CX3CR1^CRE^ TBI groups, but mice in the NLRP3^CX3CR1^ TBI group spent longer time in the center and less time in the perimeter than mice in the CX3CR1^CRE^ TBI group (Figure 5D–F).

**FIGURE 6:**
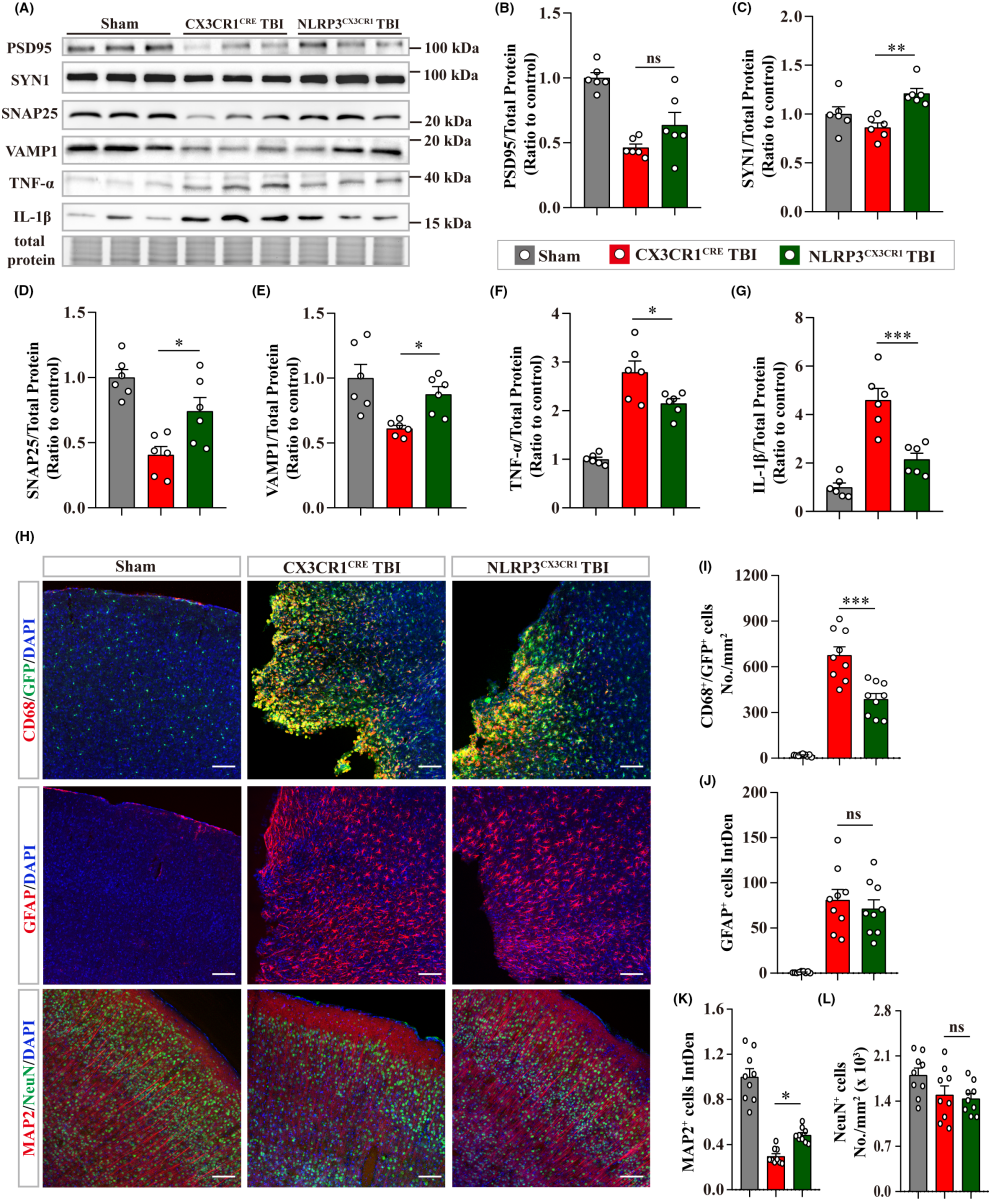
Microglial NLRP3 conditional knockout diminished neuropathology at 7‐d post‐TBI. (A) Representative immunoblots of synaptic proteins (PSD95, SYN1, SNAP25, and VAMP1), inflammatory cytokines (TNF‐α and IL‐1β), and total protein image were acquired using stain‐free technology of Bio‐Rad (a portion of full lane images were shown) at 7‐d post‐TBI. (B–G) Quantitative analysis of immunoblots (*n* = 6). (H) Representative images of immunofluorescence staining for CD68 and GFP, GFAP, MAP2, and NeuN in mouse brain slices. Group information is shown in images. Scale bar = 100 μm. (I) Quantitative analysis of CD68 and GFP double positive cells. (J, K) Quantitative analysis of the integrated intensity of GFAP^+^ and MAP2^+^ cells. (L) Quantitative analysis of the number of NeuN^+^ cells. For (I–L), three mouse brains and three randomly selected slices containing injured sites from each brain were used in each group. Data are presented as the mean ± SEM and passed the Kolmogorov–Smirnov test for normality (*p* > 0.1), and then data were analyzed using one‐way ANOVA followed by Tukey's multiple comparisons test for significance. ns: *p* > 0.05, *p < 0.05, **p < 0.01, and ***p < 0.001.

Then the effects of microglial NLRP3 conditional knockout on the neuropathology at 7‐d post‐TBI were investigated. NLRP3^CX3CR1^ TBI group showed a decrease in the loss of synaptic proteins (SYN1, SNAP25, and VAMP1, except PSD95) and a reduction in TNF‐α and IL‐1β levels in the peri‐injured cortex than CX3CR1^CRE^ TBI group (Figure 6A–G). Moreover, immunofluorescence staining revealed that microglia activation (CD68^+^ and GFP^+^) was significantly ameliorated by microglial NLRP3 conditional knockout post‐TBI, but it had no effects on the astrogliosis (GFAP^+^) (Figure 6H–J). NLRP3^CX3CR1^ TBI group also exhibited an increased fluorescence intensity of MAP2^+^ than the CX3CR1^CRE^ TBI group, but there was no significant increase in the number of neurons (NeuN^+^) in the peri‐injured cortex (Figure 6H,K,L).

### 3.6 Microglial A_2A_R conditional knockout reduced NLRP3 inflammasome activation and mitigated the neurological deficits and neuropathology post‐TBI

**FIGURE 7:**
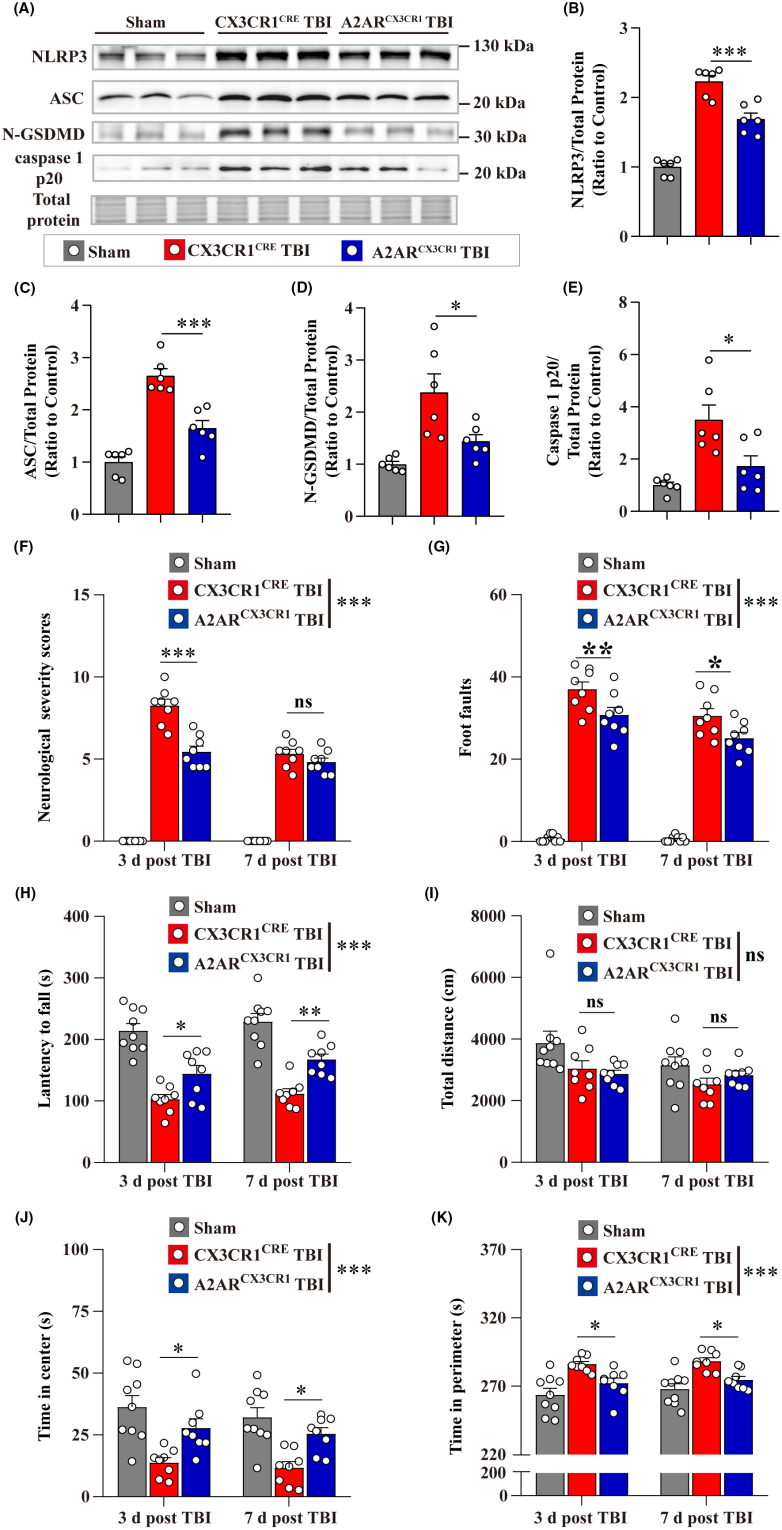
Microglial A_2A_R conditional knockout attenuated NLRP3 inflammasome activation and neurological deficits post‐TBI. (A) Representative images of immunoblots for NLRP3, ASC, N‐GSDMD, caspase 1 p20, and total protein images were acquired with stain‐free technology of Bio‐Rad (a portion of full lane images were shown) at 7‐d post‐TBI. (B–E) Quantitative analysis of NLRP3, ASC, N‐GSDMD, and caspase 1 p20 levels (*n* = 6). (F–H) The results of the NSS score, beam walk test, and accelerating rotarod test at 3‐ and 7‐d post‐TBI (*n* = 8–10). (I–K) The total distance traveled, time spent in center, and time spent in perimeter were recorded in the open field test at 3‐ and 7‐d post‐TBI (*n* = 8–10). Data are presented as the mean ± SEM and passed the Kolmogorov–Smirnov test for normality (*p* > 0.1), and then data were analyzed using two‐way ANOVA followed by Tukey's multiple comparisons test for comparison at each time point. ns: *p* > 0.05, *p < 0.05, **p < 0.01, and ***p < 0.001.

We then tested the effects of microglial A_2A_R on the NLRP3 inflammasome activation post‐TBI using microglial A_2A_R conditional knockout mice (A_2A_R^CX3CR1^ mice) and matched littermate control CX3CR1^CRE^ mice (used in the sham and CX3CR1^CRE^ TBI group). Our results showed that levels of NLRP3, ASC, N‐GSDMD, and caspase 1 p20 proteins were significantly decreased in the A_2A_R^CX3CR1^ TBI group than that in CX3CR1^CRE^ TBI group at 7‐d post‐TBI, indicating that microglial A_2A_R conditional knockout attenuated NLRP3 inflammasome activation postinjury (Figure 7A–E).

Then behavioral tests were performed at 3‐ and 7‐d post‐TBI to assess the effects of microglial A_2A_R knockout on neurological deficits. There are no significant differences between A_2A_R^CX3CR1^ mice and their CX3CR1^CRE^ littermates in behavioral tests used in the article before TBI (Figure S4E–H). Our results demonstrated that the A_2A_R^CX3CR1^ TBI group exhibited lower NSS scores, fewer foot faults in the beam walk test, and longer latency to fall in accelerating rotarod test than CX3CR1^CRE^ TBI group (Figure 7F–H). In the open field test, no significant differences were found in the total distance between A_2A_R^CX3CR1^ TBI and CX3CR1^CRE^ TBI group, but mice in the A_2A_R^CX3CR1^ TBI group spent a longer time in the center and less time in the perimeter than mice in the CX3CR1^CRE^ TBI group (Figure 7I–K).

**FIGURE 8:**
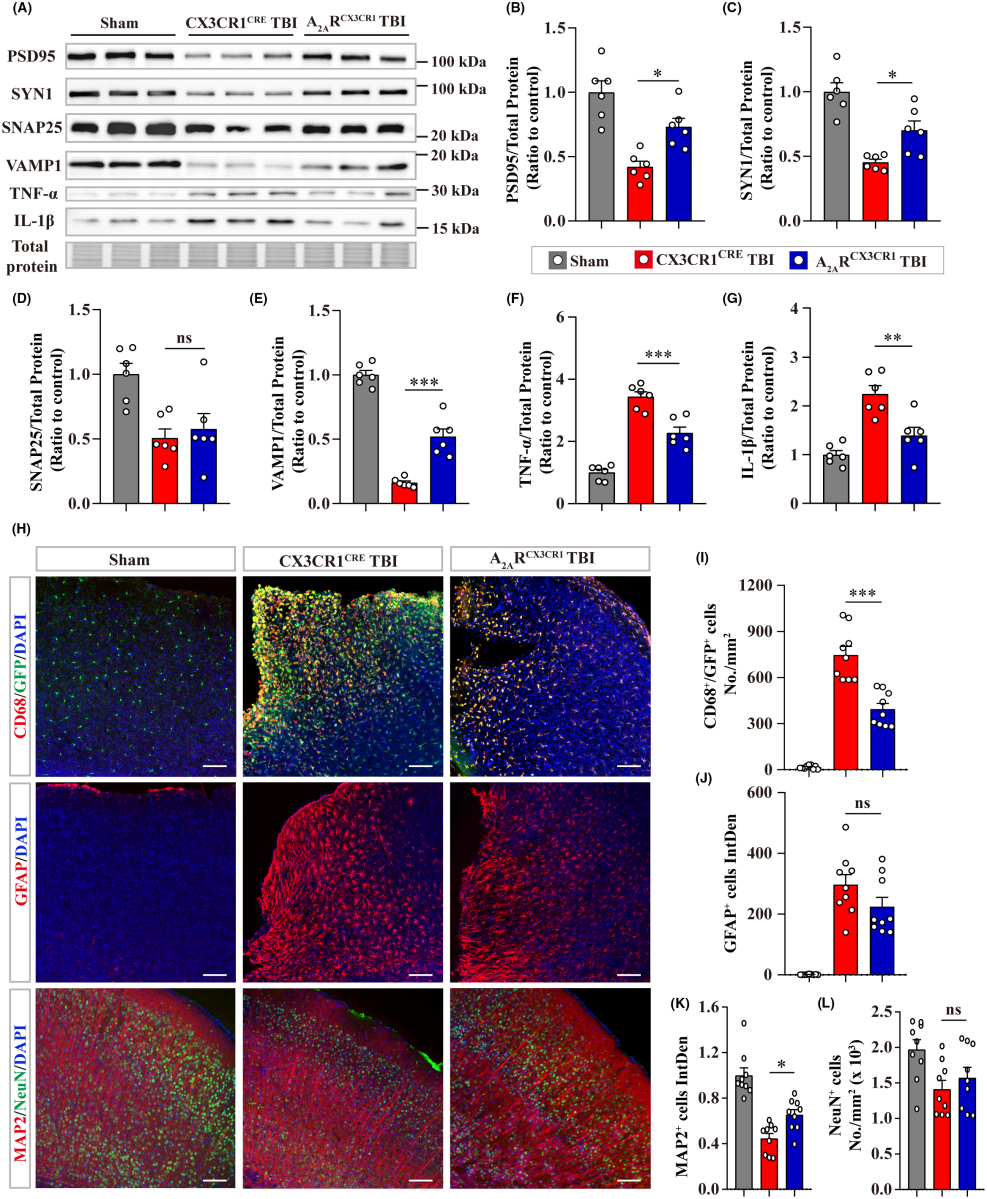
Microglial A_2A_R conditional knockout mitigated neuropathology at 7‐d post‐TBI. (A) Representative immunoblots of synaptic proteins (PSD95, SYN1, SNAP25, and VAMP1), inflammatory cytokines (TNF‐α and IL‐1β), and total protein images acquired with stain‐free technology of Bio‐Rad (a portion of full lane images were shown) at 7‐d post‐TBI. (B–G) Quantitative analysis of the immunoblots shown above (*n* = 6). (H) Representative images of immunofluorescence staining for CD68, GFAP, MAP2, and NeuN. Scale bar = 100 μm. (I) Quantitative analysis of CD68 and GFP double‐positive cells. (J, K) Quantitative analysis of the integrated intensity of GFAP^+^ and MAP2^+^ cells. (L) Quantitative analysis of the number of NeuN^+^ cells. For (I–L), three mouse brains and three randomly selected slices containing injured sites from each brain were used in each group. Data are presented as the mean ± SEM and passed the Kolmogorov–Smirnov test for normality (*p* > 0.1), and then data were analyzed using one‐way ANOVA followed by Tukey's multiple comparisons test for significance. ns: *p* > 0.05, *p < 0.05, **p < 0.01, and ***p < 0.001.

The effects of microglial A_2A_R conditional knockout on the neuropathology at 7‐d post‐TBI were investigated. A significant increase in the levels of synaptic proteins (PSD95, SYN1, and VAMP1, except SNAP25) and a reduction in the levels of TNF‐α and IL‐1β were detected in the peri‐injured cortex of A_2A_R^CX3CR1^ TBI group compared with CX3CR1^CRE^ TBI group (Figure 8A–G). Microglial activation (CD68^+^ and GFP^+^) was attenuated by microglial A_2A_R knockout post‐TBI with no significant difference in the astrogliosis (GFAP^+^) (Figure 8H–J). In the A_2A_R^CX3CR1^ TBI group, the fluorescence intensity of MAP2^+^ cells was significantly elevated, while the number of NeuN^+^ cells did not change compared with that in the CX3CR1^CRE^ TBI group (Figure 8H,K–L).

## 4 DISCUSSION

A_2A_R is widely expressed in both peripheral and central nervous systems and plays a crucial role in the regulation of physical and pathological processes in diverse diseases.^36^ However, A_2A_R exerted opposite effects on the peripheral and central nervous systems. A_2A_R activation in the peripheral system usually showed anti‐inflammatory effects and A_2A_R agonists had beneficial effects on asthma and wound healing.^37^, ^38^ In contrast, A_2A_R inhibition revealed neuroprotective effects in many CNS diseases including TBI, AD, and PD.^39^, ^40^, ^41^, ^42^ Our group had reported that the opposite effects of A_2A_R were associated with the extracellular glutamate concentrations.^12^, ^13^ A_2A_R interacted with metabotropic glutamate receptor 5 (mGluR5) and the high glutamate concentration redirected the downstream signaling pathway of A_2A_R from anti‐inflammatory protein kinase A (PKA) to pro‐inflammatory protein kinase C pathway. Similar effects were also found in the regulation of A_2A_R on NLRP3 inflammasome in microglia.^27^ Moreover, it is reported that both A_2A_R agonists and antagonists protect against spinal cord injury, as explained by the different effects of A_2A_R on the peripheral system and CNS.^43^ Therefore, the effects of A_2A_R in peripheral system diseases may not be the same as that in CNS diseases. In addition, A_2A_R is expressed in both neurons and glial cells in the CNS and plays different roles in these cells.^11^, ^44^ Thus, the roles of A_2A_R in diverse types of cells and diseases should be elucidated for a better understanding of the underlying mechanisms and for finding more effective therapeutic targets. Previous studies have shown that A_2A_R was involved in regulating the NLRP3 inflammasome in macrophages and endothelial cells.^10^, ^15^, ^16^ But the effects of A_2A_R on the NLRP3 inflammasome in the CNS remain to be elucidated. Here, we demonstrated that microglial A_2A_R conditional knockout inhibited the NLRP3 inflammasome activation and attenuated the neuroinflammation post‐TBI. Our findings contribute to a better understanding of the important roles of microglial A_2A_R in neuroinflammation regulation.

In addition, we proved that A_2A_R could interact with NLRP3 and impact the NLRP3 inflammasome assembly and activation. The inhibition of this interaction mitigated NLRP3 inflammasome activation‐induced pyroptosis and release of inflammatory cytokines, which contributed to the attenuation of neuroinflammation post‐TBI. Our results provided a novel mechanism for the regulation of NLRP3 inflammasome in the CNS. Actually, A_2A_R generally serves as a neuromodulator in the CNS,^45^ and its effects were also reported to require the cooperation of other receptors, including the adenosine A1 receptor, adenosine A3 receptor, and NMDA receptor.^46^, ^47^, ^48^, ^49^ Moreover, it is found recently that NLRP3 forms a double‐ring cage, and this structure is primarily membrane localized, which provided the structural basis for the interaction between NLRP3 and A_2A_R.^50^ But more research are needed to uncover the interaction motifs between NLRP3 and A_2A_R.

The NLRP3 inflammasome is an important regulator of the innate immune system and participates in many pathological processes in CNS diseases including TBI, AD, and PD.^5^, ^51^ A lot of efforts have been made to better understand the molecular mechanisms of NLRP3 inflammasome activation in order to find potential therapeutic targets. Abundant inhibitors of NLRP3 inflammasome have been reported to date, including those that either directly target NLRP3 inflammasome components or related signaling pathways. However, some small molecule drugs that directly target NLRP3 inflammasome components, such as NLRP3 inhibitor MCC950 and caspase 1 inhibitor VX‐740 and VX‐765, failed to pass the clinical trial because of hepatic toxicity.^6^, ^52^ Some studies also tried to use drugs targeting other receptors or signaling pathways to inhibit the NLRP3 inflammasome activation, which means to find new applications for old drugs.^53^, ^54^, ^55^, ^56^ These studies provided an alternative way to promote the clinical application of drugs for the treatment of NLRP3 inflammasome‐related diseases. However, NLRP3 inflammasome could be activated by abundant damage‐associated molecular patterns and pathogen‐associated molecular patterns so the indirect inhibition to one of the activation pathways may not be effective enough and the off‐target effects of these drugs should also be considered carefully. Therefore, it is important to find a safe and effective enough target for the treatment of NLRP3 inflammasome‐related diseases. Here we reported that A_2A_R could regulate the NLRP3 inflammasome assembly and activation by directly interacting with NLRP3 in microglia. The inhibition of this interaction by pharmacological treatment or knockout attenuated the inflammasome activation and contributed to the reduction of neuroinflammation post‐TBI. This direct regulation of the NLRP3 inflammasome assembly and activation might be more effective than targeting signaling pathways involved in the priming or activating steps of the inflammasome activation. Moreover, the inhibition of A_2A_R revealed a broad range of neuroprotective effects in diverse CNS diseases.^57^ Thus, there might be less off‐target effects and more benefits when using A_2A_R antagonists in the treatment of CNS diseases. Because of the well‐developed drugs‐targeting A_2A_R including istradefylline and regadenoson,^39^, ^58^ which have been proved by the US Food and Drug Administration for clinical therapy, A_2A_R may be an ideal target for the treatment of NLRP3 inflammasome‐related CNS diseases.

The NLRP3 inflammasome is also reported to be expressed in neurons, astrocytes, and oligodendrocytes in addition to microglia, where it contributes to synaptic damage and myelin degeneration.^59^, ^60^, ^61^ These diverse distributions and effects of NLRP3 inflammasome urge more efforts to interrogate the role of it in different cell types and diseases. In our study, we investigated the role of microglial NLRP3 inflammasome in neurological deficits and neuropathology post‐TBI. Moreover, we found that microglial NLRP3 conditional knockout mainly showed neuroprotective effects on neural dendrites and axons (MAP2^+^) rather than the apoptosis of neurons (no significant changes on NeuN^+^ cells), which provided a different downstream effect of microglial NLRP3. This study contributes to elucidating the significant roles and underlying pathological effects of microglial NLRP3 inflammasome activation in CNS diseases.

In general, our findings demonstrated a novel mechanism for A_2A_R to regulate the NLRP3 inflammasome assembly and activation, which further contributed to the neuroinflammation regulation post‐TBI. Because of the well‐developed A_2A_R‐targeted drugs including istradefylline and regadenoson,^39^, ^58^ A_2A_R may be an ideal target for the treatment of NLRP3 inflammasome‐related CNS diseases.

## AUTHOR CONTRIBUTIONS

Hao Du: Conceptualization, Methodology, Investigation, Writing—original draft preparation; Chang‐Hong Li: Formal analysis, Data curation, Writing—review and editing; Ruo‐Bing Gao: Formal analysis, Data curation; Yan Tan: Formal analysis, Data curation; Bo Wang: Investigation, Data curation; Yan Peng: Investigation, Data curation; Ya‐Lei Ning: Conceptualization, Supervision, Project administration; Ping Li: Conceptualization, Supervision, Project administration; Yan Zhao: Conceptualization, Supervision, Project administration, Writing—review and editing; Yuan‐Guo Zhou: Conceptualization, Supervision, Project administration, Writing—review and editing, Funding acquisition.

## FUNDING INFORMATION

This study was supported by the National Natural Science Foundation of China (grant no. 31771118).

## CONFLICT OF INTEREST STATEMENT

The authors declare that they have no competing interests.

## CONSENT FOR PUBLICATION

The study did not involve human subjects, so there is no consent to participate.

## Supporting information


Appendix S1

## Data Availability

The data and materials that support the findings of this study are available from the corresponding author upon reasonable request.
