# Supplementary material for: Inhibition of the interaction between microglial adenosine 2A receptor and NLRP3 inflammasome attenuates neuroinflammation posttraumatic brain injury
Source: CNS Neurosci Ther. 2023 Aug 11;30(1):e14408. doi: 10.1111/cns.14408 (PMC10805470; doi:10.1111/cns.14408)

**Table S1. Primers for identification of transgene mice.**

| mice | primers | Expected results |
| --- | --- | --- |
| A_2A_R KO | P1: AGCCAGGGGTTACATCTGTG | Wild type= ~180bp  Heterozygote= ~180bp and ~320bp  Homozygote= ~320bp |
|  | P2: TACAGACAGCCTCGACATGTG |  |
|  | P3: TCGGCCATTGAACAAGATGG |  |
|  | P4: GAGCAAGGTGAGATGAGAGG |  |
| NLRP3 KO | P1: TCAGTTTCCTTGGCTACCAGA | Wild type= ~666bp  Heterozygote= ~666bp and ~850bp  Homozygote= ~850bp |
|  | P2: TTCCATTACAGTCACTCCAGATGT |  |
|  | P3: TGCCTGCTCTTTACTGAAGG |  |
| A_2A_R-flox | P1: TGAGAAGGGTTGGGTGAAGAT | Wild type= ~146bp |
|  | P2: GCCACTTCAGATTTTGTGTCC | Heterozygote= ~146bp and ~186bp  Homozygote= ~186bp |
| NLRP3- flox | P1: CATCCACAGGAGTATGCCCATG | Wild type= ~305bp |
|  | P2: CTTCAGAAGGGAACATCGTCAGTC | Heterozygote= ~305bp and ~410bp  Homozygote= ~410bp |
| CX3CR1^CRE/ERT2^ | P1: AAGACTCACGTGGACCTGCT | Wild type= ~695bp |
|  | P2: CGGTTATTCAACTTGCACCA | Heterozygote= ~300bp and ~695bp  Homozygote= ~300bp |
|  | P3: AGGATGTTGACTTCCGAGTTG |  |

**Table S2. Antibodies and the dilution used in the article.**

| application | primary antibody | dilution | company | catalog number |
| --- | --- | --- | --- | --- |
| WB | anti-NLRP3 | 1:1000 | Adipogen | AG-20B-0014 |
|  | anti-ASC | 1:1000 | CST | 67824 |
|  | anti-caspase 1 | 1:1000 | Adipogen | AG-20B-0042 |
|  | anti-caspase 1 p20 | 1:1000 | CST | 89332 |
|  | anti-GSDMD | 1:1000 | Abcam | ab209845 |
|  | anti-N-terminal of GSDMD | 1:1000 | CST | 10137 |
|  | anti-A_2A_R | 1:200 | Santa Cruz | sc-32261 |
|  | anti-IL-1β | 1:1000 | Abcam | ab200478 |
|  | anti-TNF-α | 1:1000 | Abcam | ab215188 |
| IF | anti-ASC | 1:800 | CST | 67824 |
|  | anti-NLRP3 | 1:200 | Adipogen | AG-20B-0014 |
|  | anti-A2AR | 1:200 | Frontier institute | AB-2571655 |
|  | anti-CD68 | 1:500 | Abcam | ab125212 |
|  | anti-GFAP | 1:250 | Abcam | ab7260 |
|  | anti-MAP2 | 1:500 | Abcam | ab32454 |
|  | anti-NeuN | 1:100 | Millipore | MAB-377 |
|  | anti-GFP | 1:1000 | Abcam | ab13970 |

**Table S3. Primers used for quantitative PCR**

| Gene | Primers |
| --- | --- |
| NLRP3 | Forward: GCCGTCTACGTCTTCTTCCTTTCC |
|  | Reverse: CATCCGCAGCCAGTGAACAGAG |
| ASC | Forward: GGACGGAGTGCTGGATGCTTTG |
|  | Reverse: CATCTTGTCTTGGCTGGTGGTCTC |
| caspase 1 | Forward: ACAACCACTCGTACACGTCTTGC |
|  | Reverse: CCAGATCCTCCAGCAGCAACTTC |
| GSDMD | Forward: ACTGAGGTCCACAGCCAAGAGG |
|  | Reverse: GCCACTCGGAATGCCAGGATG |
| IL-1β | Forward: TCGCAGCAGCACATCAACAAGAG |
|  | Reverse: AGGTCCACGGGAAAGACACAGG |
| IL-18 | Forward: CAAAGTGCCAGTGAACCCCAGAC |
|  | Reverse: ACAGAGAGGGTCACAGCCAGTC |
| GAPDH | Forward: CTTTGTCAAGCTCATTTCCTGG |
|  | Reverse: TCTTGCTCAGTGTCCTTGC |

**Table S4. The number of mice used in each group for different tests.**

| **Experiments** | **The number of mice used in sham groups** | | **The number of mice used in TBI groups** | | | |
| --- | --- | --- | --- | --- | --- | --- |
|  | **WT** | **CX3CR1CRE** | **WT** | **CX3CR1^CRE^** | **A_2A_R^CX3CR1^** | **NLRP3^CX3CR1^** |
| WB (sorted microglia) | 9 | / | 9 | / | / | / |
| WB (brain tissue) | / | 12 | / | 12 | 6 | 6 |
| Neurobehavioral tests | / | 20 | / | 20 | 10 | 10 |
| Immunofluorescence staining | 3 | 6 | 3 | 6 | 3 | 3 |
| Note: Mice used for neurobehavioral tests were sacrificed finally for WB and immunofluorescence staining. | | | | | | |

**Figure S1. Representative results of agarose gel electrophoresis for transgenic mice genotyping.** (**A-E**) The representative results of A_2A_R-flox, NLRP3-flox, CX3CR1^CRE^, A_2A_R-KO and NLRP3-KO mice genotyping, respectively.


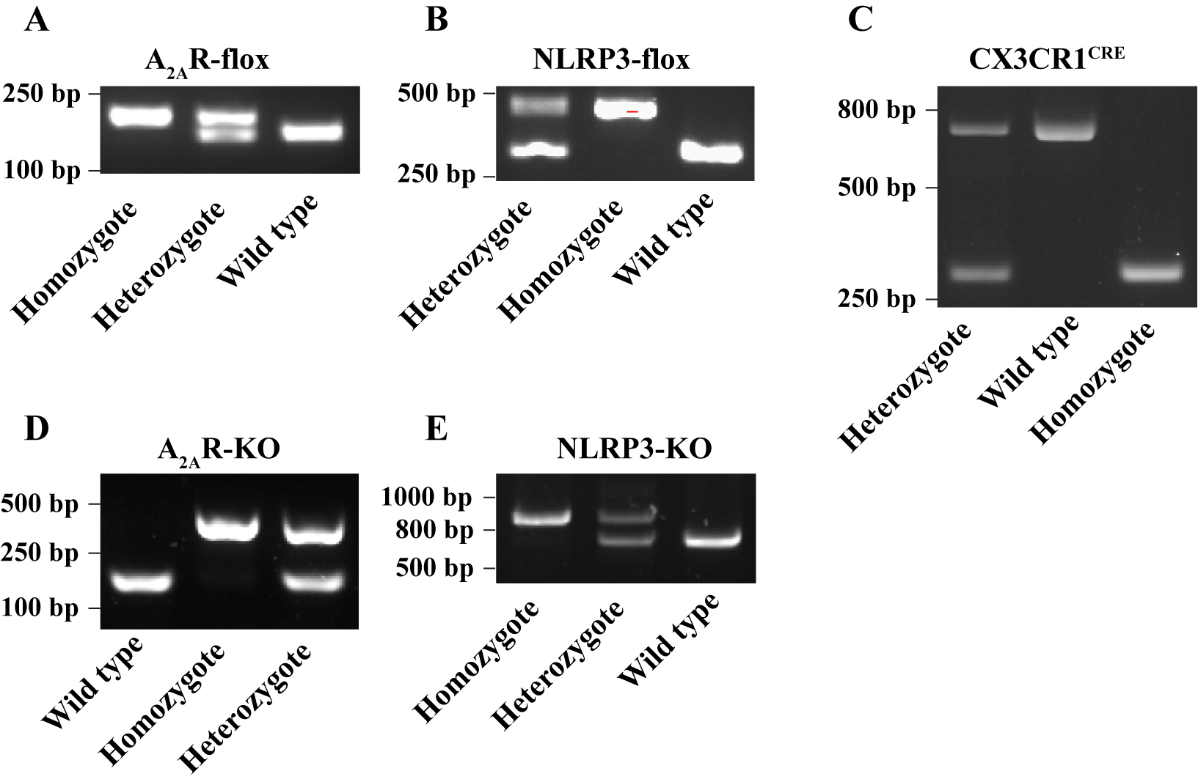


**Figure S2.** **The verification of microglial A2AR and NLRP3 conditional knockout efficiency.** MACS sorted microglia from microglial A2AR or NLRP3 conditional knockout mice and the control CX3CR1^CRE^ littermates were used to perform WB detection to ensure the efficiency of knockout.


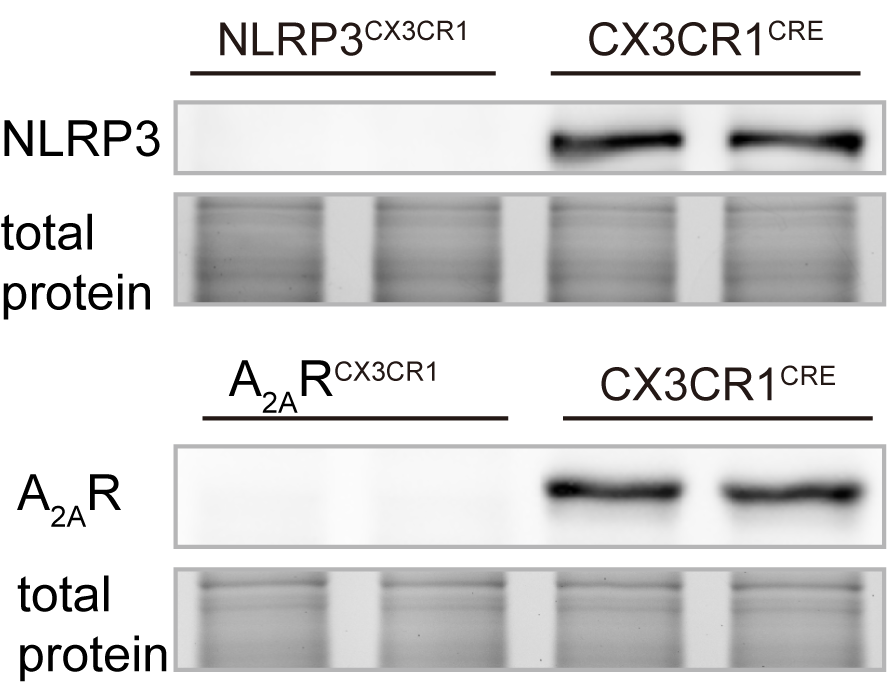


**Figure S3. TBI induced the lack of MAP2 positive and NeuN positive cells proximately to the injury area.** There are about 300 μm far from injury site that lacking apparent MAP2 positive and NeuN positive cells in all TBI treated mice. Therefore, images 300 μm from the injury site were acquired to investigate the effects of TBI on dendrites and neurons. Scale bar = 100 μm.


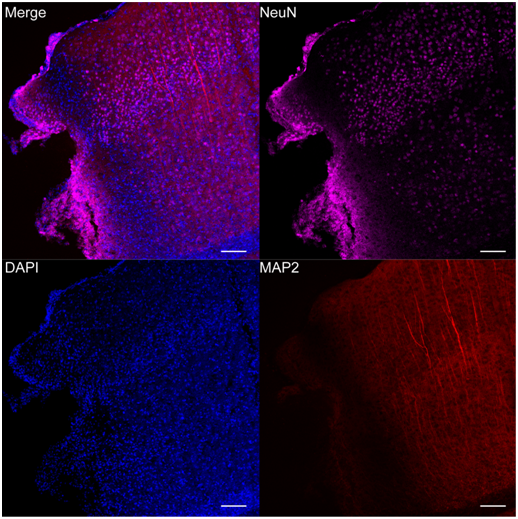


**Figure S4. The behavioral test results of A_2A_R^CX3CR1^ mice, NLRP3^CX3CR1^ and paired control CX3CR1^CRE^ mice.** (**A**) Rotarod test results of NLRP3^CX3CR1^ and paired control CX3CR1^CRE^ mice. (**B-D**) Open field test results including total distance (cm), time in center (s), and time in perimeter (s) of NLRP3^CX3CR1^ and paired control CX3CR1^CRE^ mice. (**E**) Rotarod test results for A_2A_R^CX3CR1^ and paired control CX3CR1^CRE^ mice. (**F-H**) Open field test results including total distance (cm), time in center (s), and time in perimeter (s) of A_2A_R^CX3CR1^ and paired control CX3CR1^CRE^ mice.


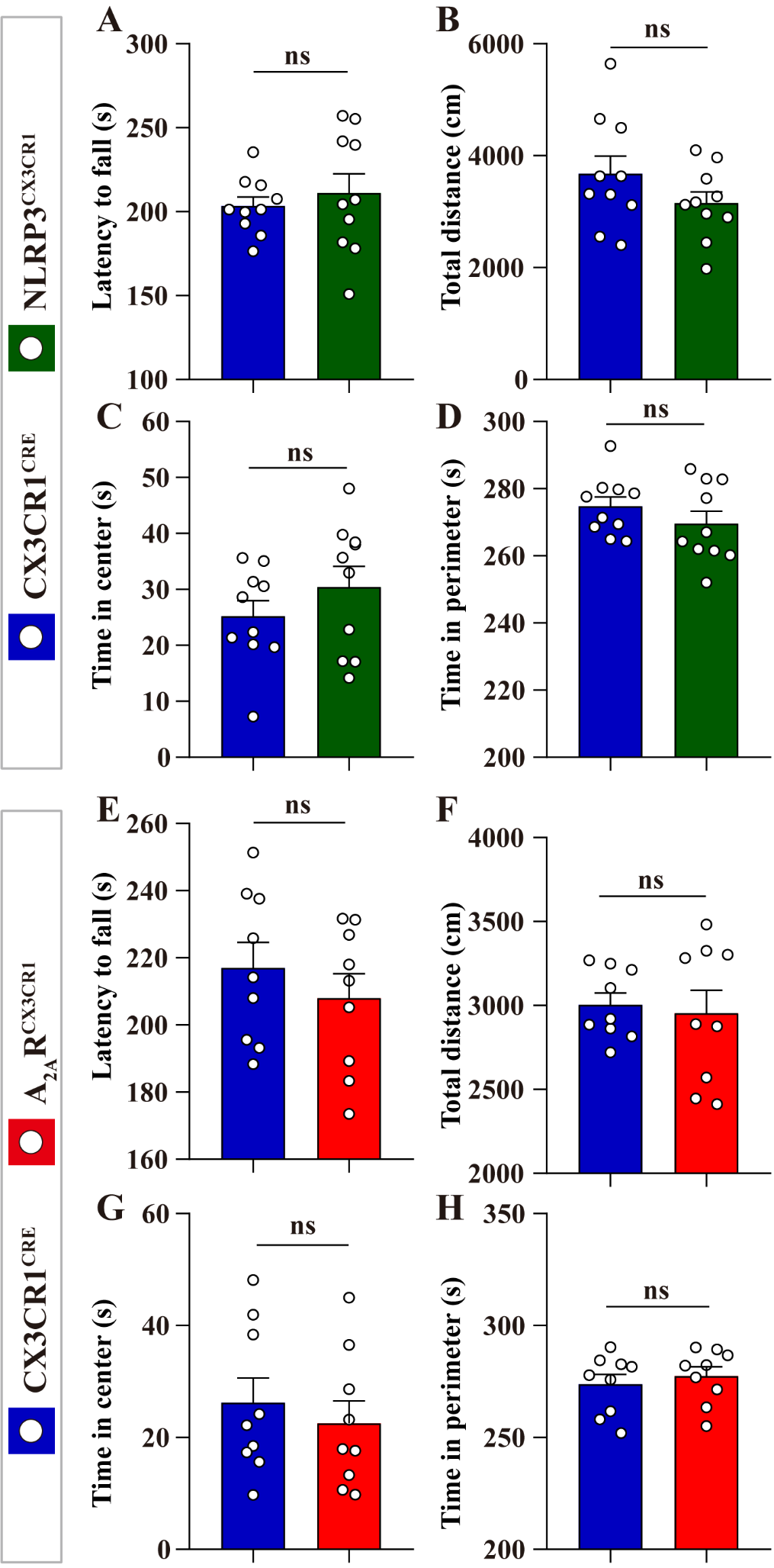


**
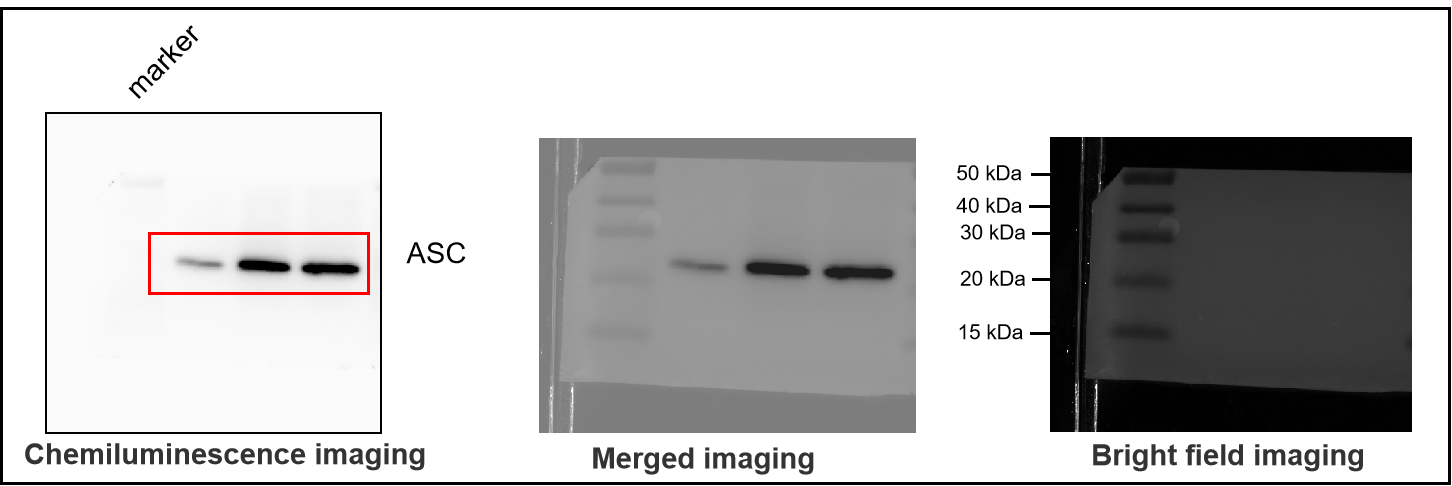
Full unedited gels/blots for Figure 2A**

The red box indicated images used in the article


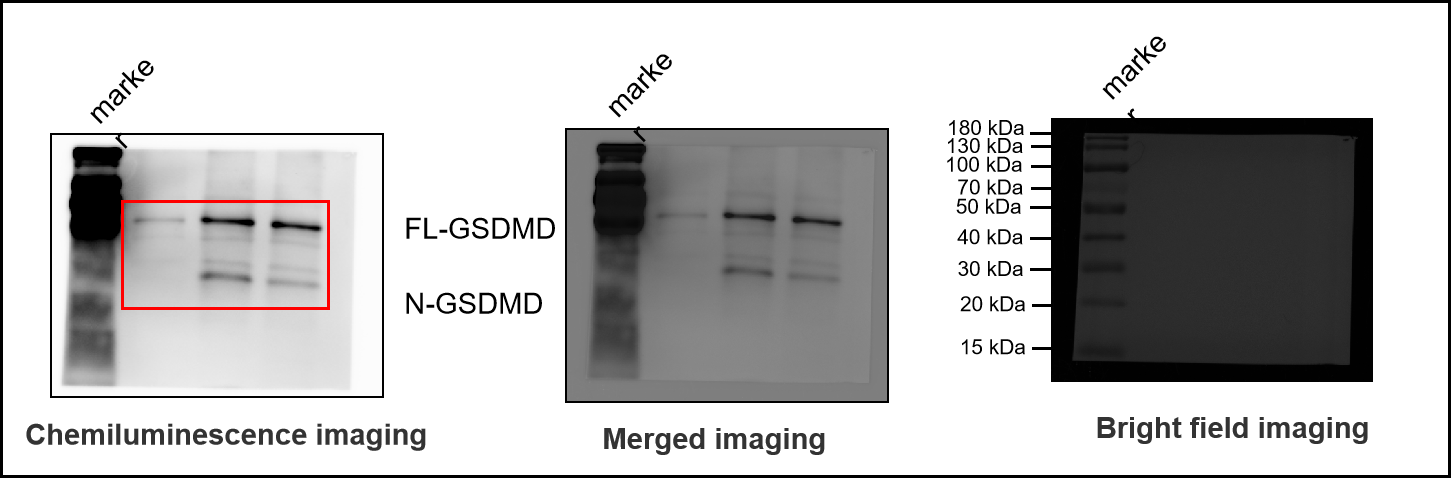


Note: Some bands of marker are not very clearly in the blot images because we chose the optimal membrane transferring time and exposure time for targeted bands. And the bright field images were acquired and merged with chemiluminescence images under this circumstance.


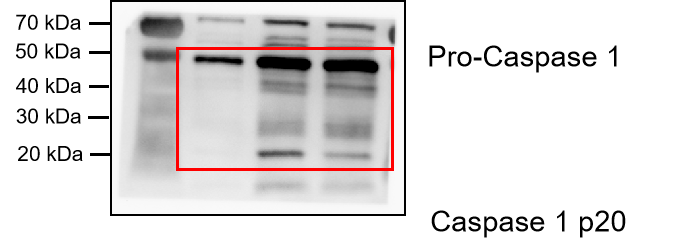

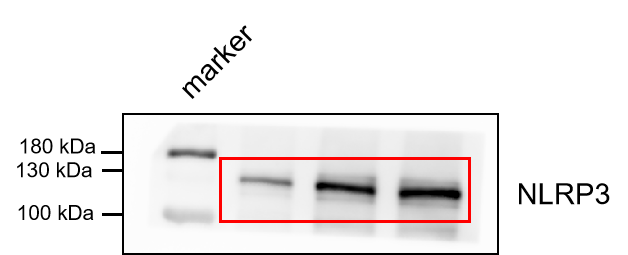

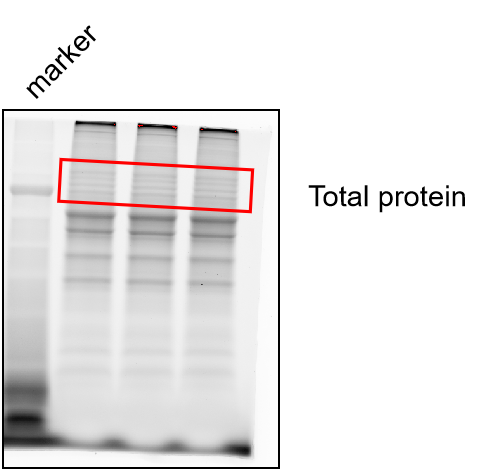


**
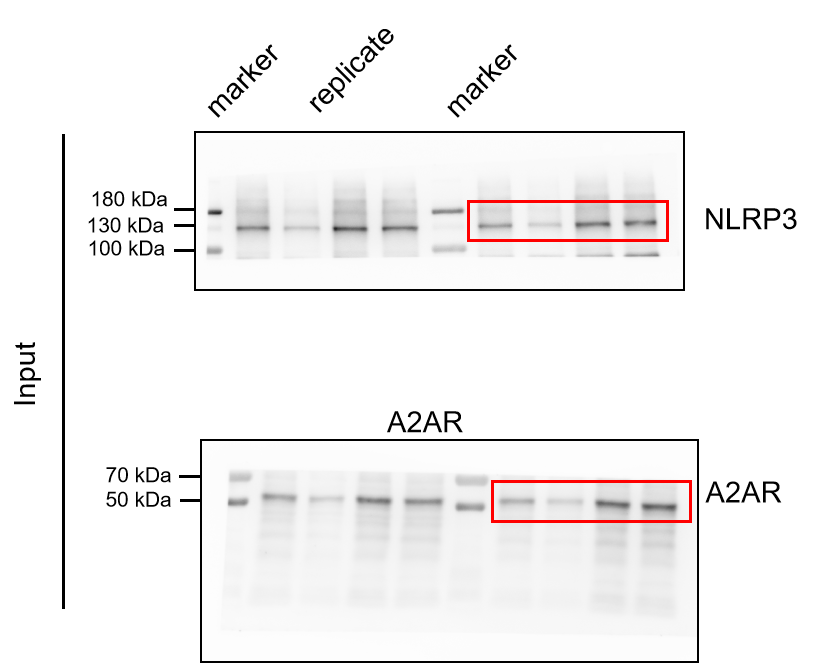

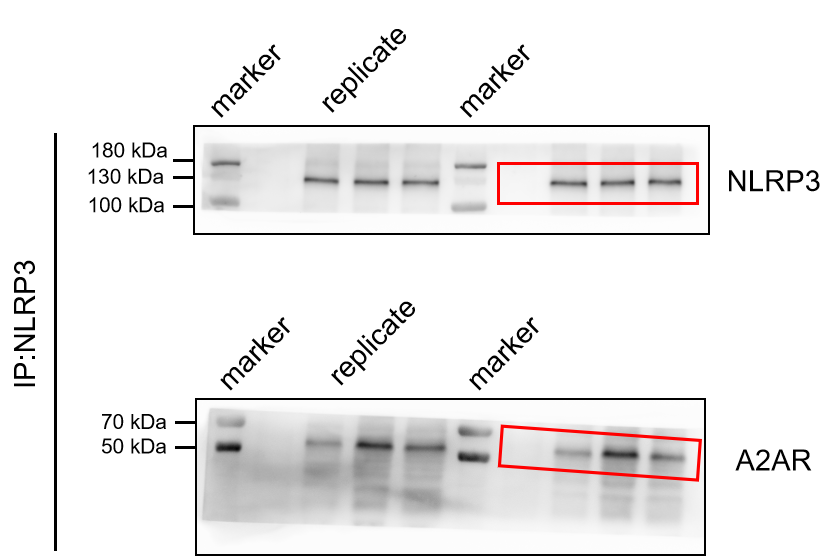
Full unedited gels/blots for Figure 3A**
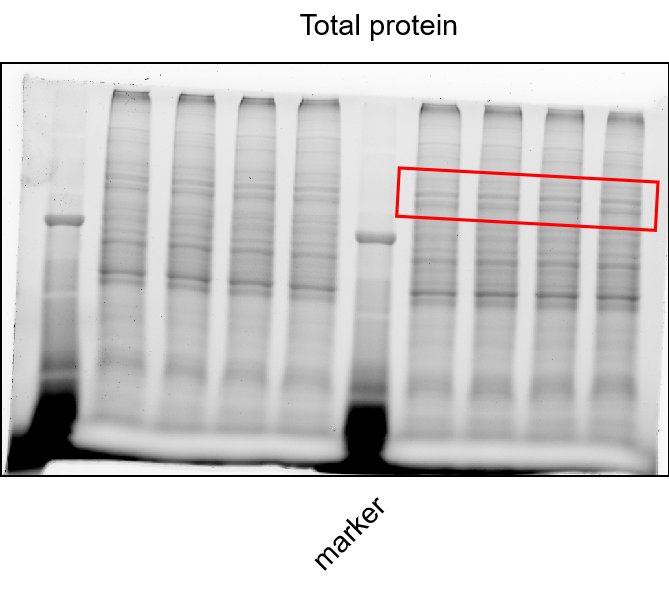


**
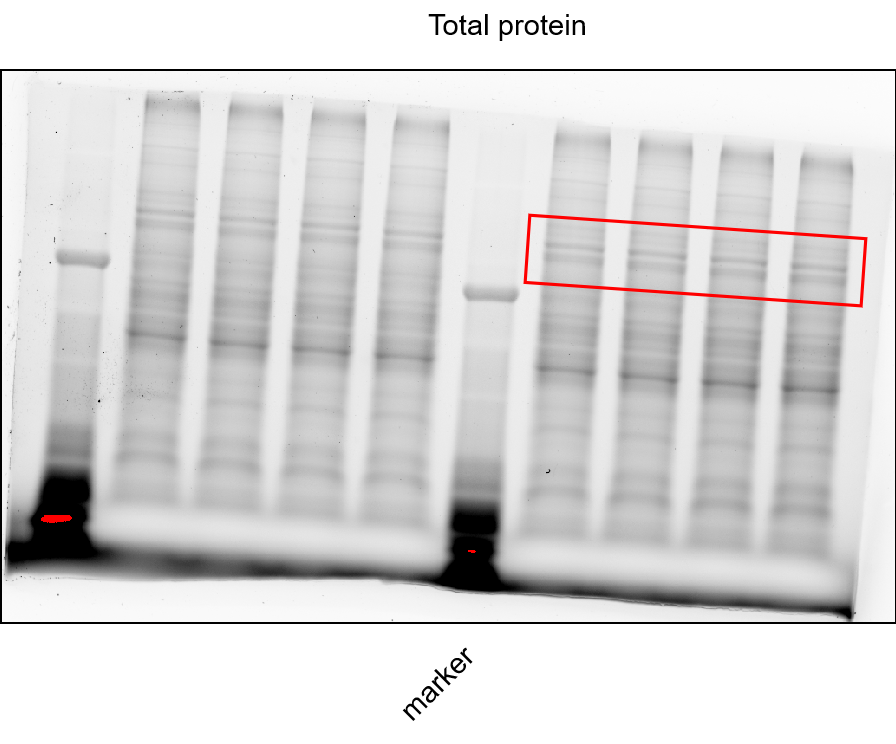

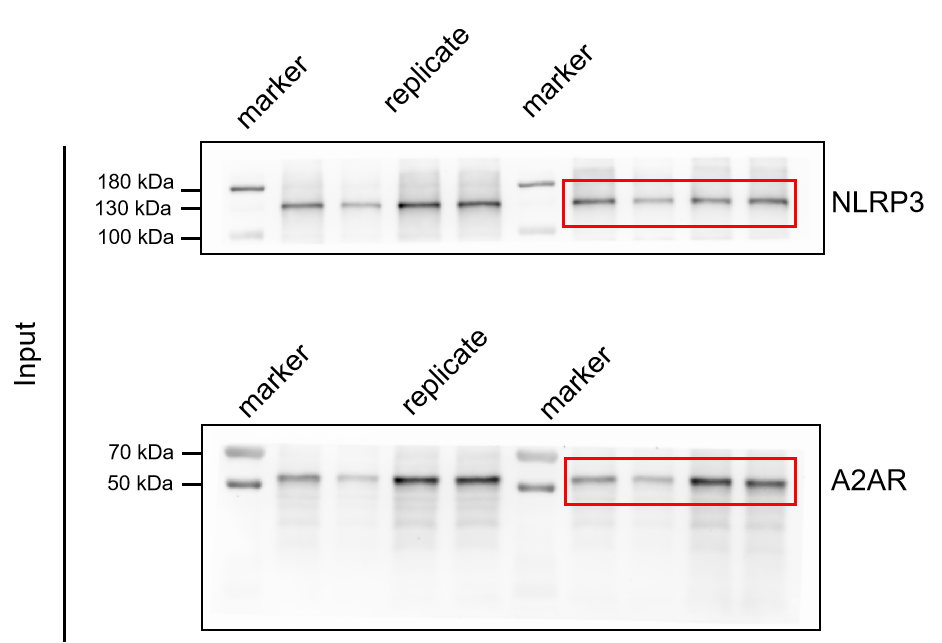

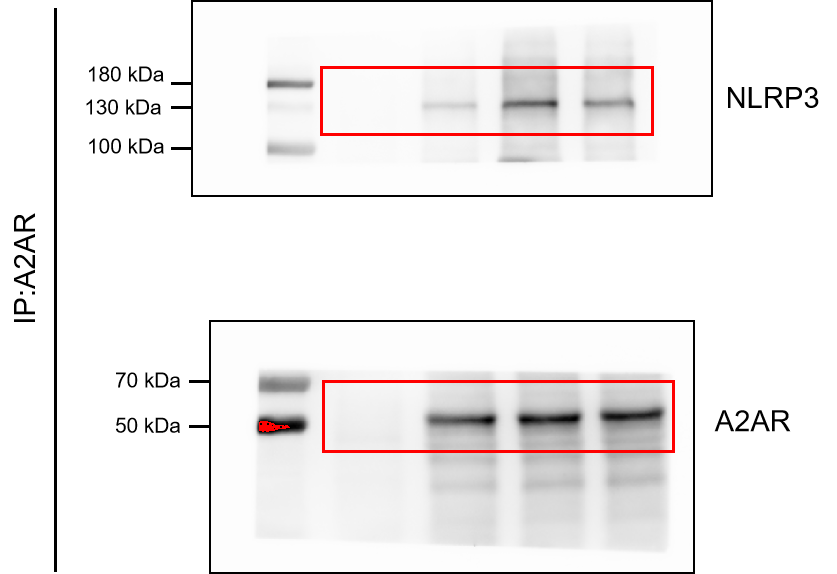
Full unedited gels/blots for Figure 3B**

**
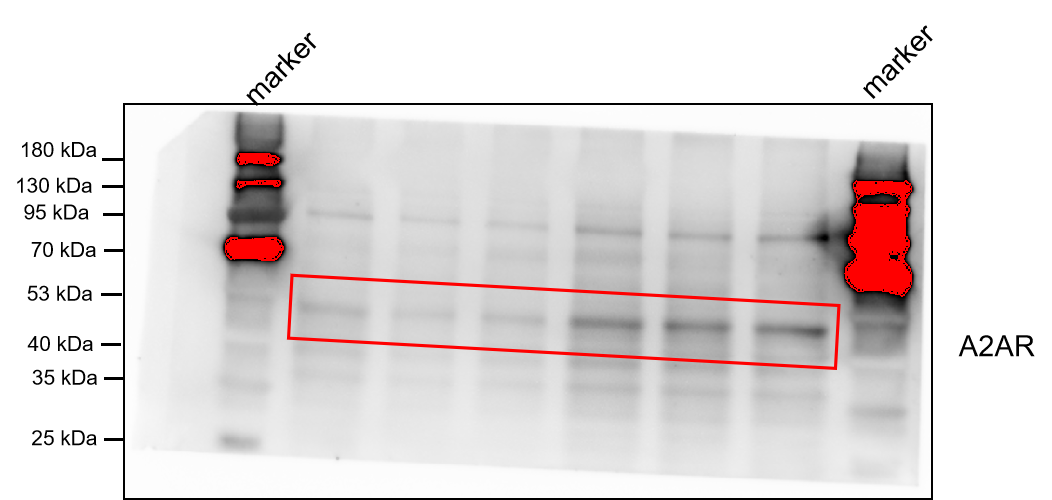

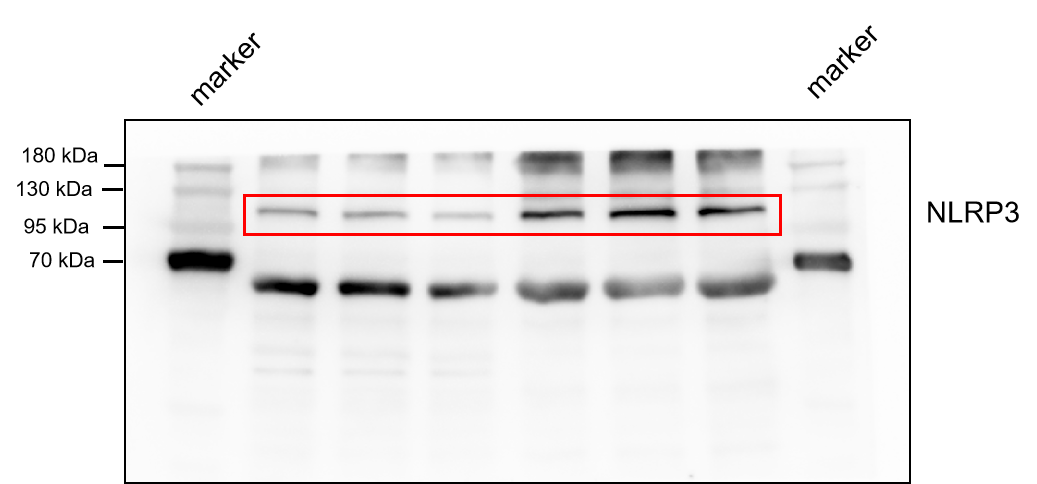
Full unedited gels/blots for Figure 4C**


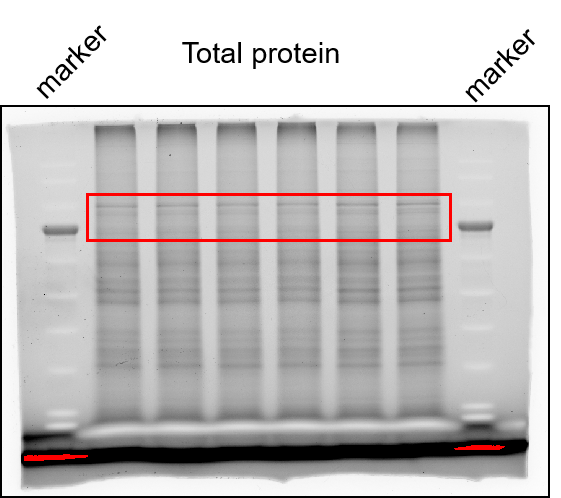
Note: Some bands of marker are red in the image because the imaging system would distinguish the oversaturated bands and areas automatically and marked it as red. We chose the optimal exposure time for the targeted bands so other areas might be over exposed sometimes.

**
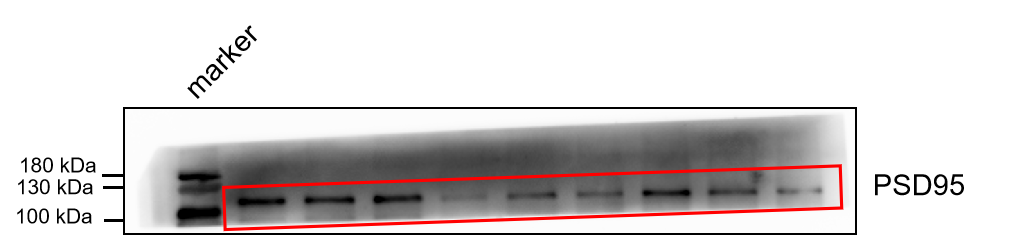

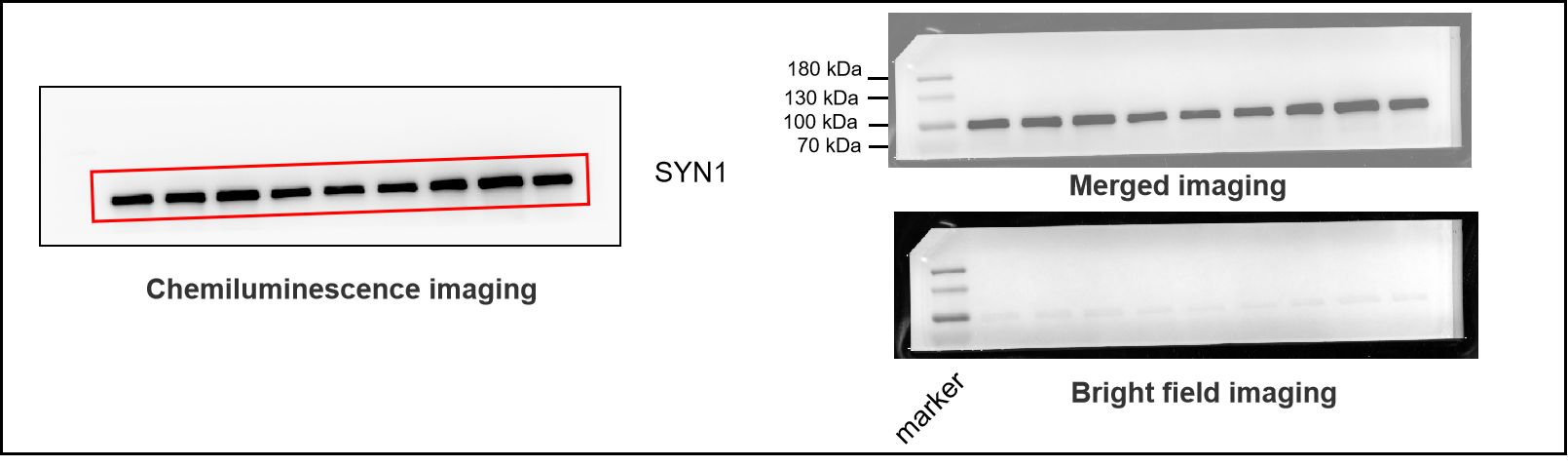

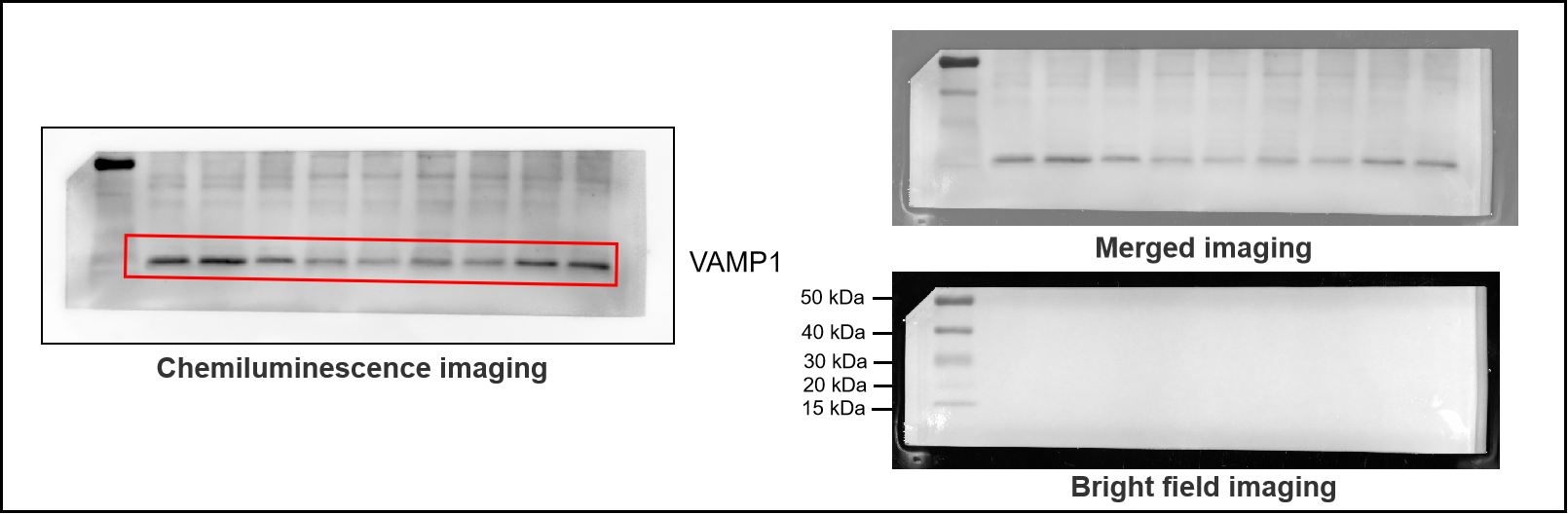

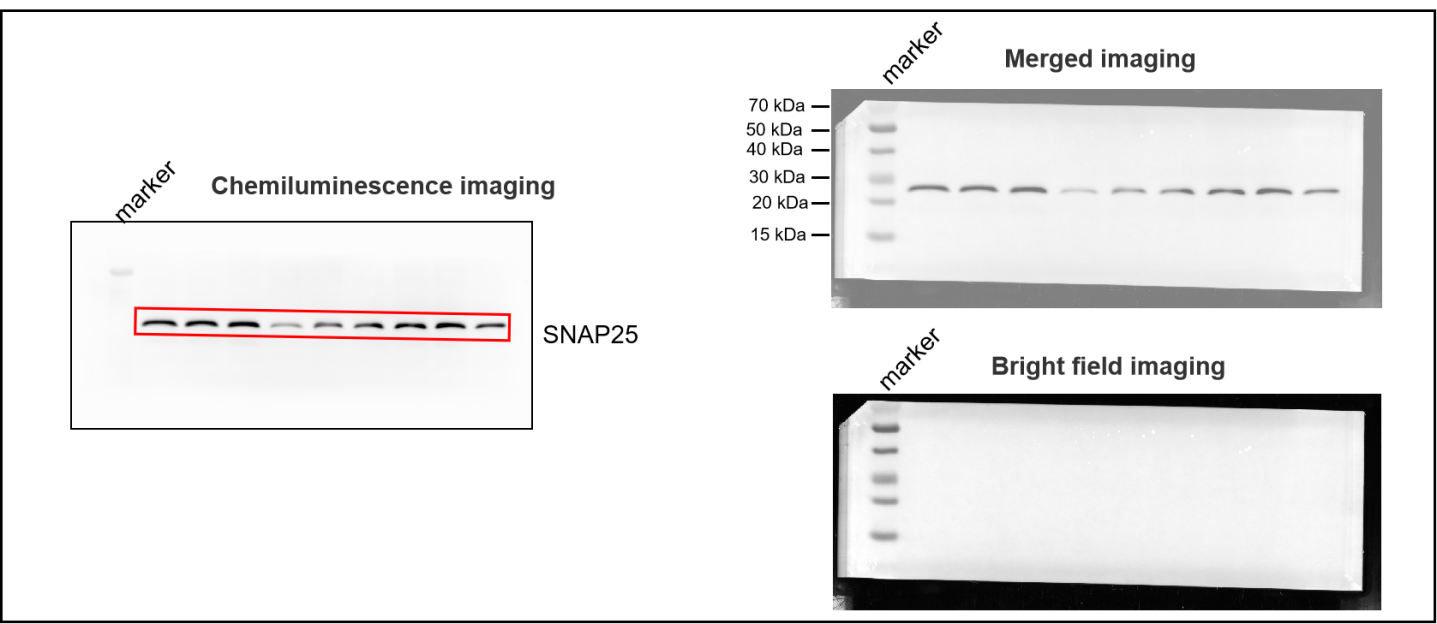
Full unedited gels/blots for Figure 6A**


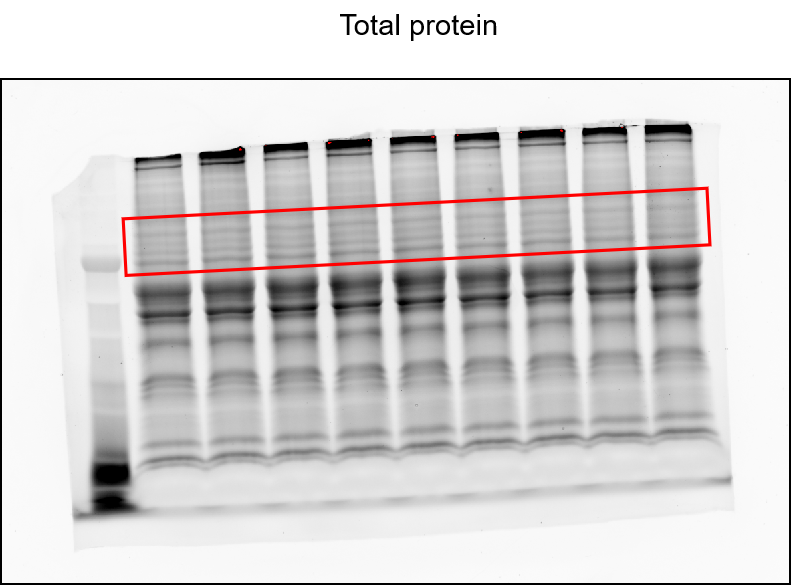

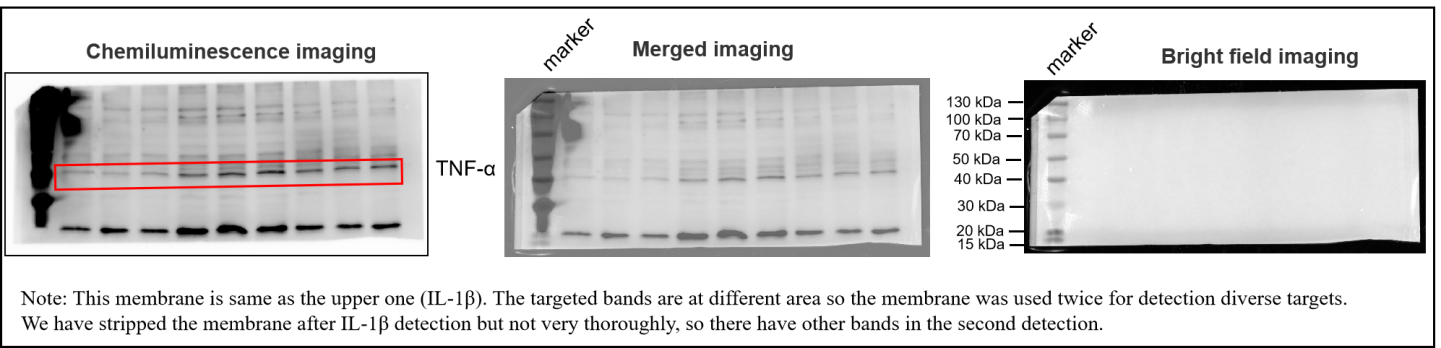

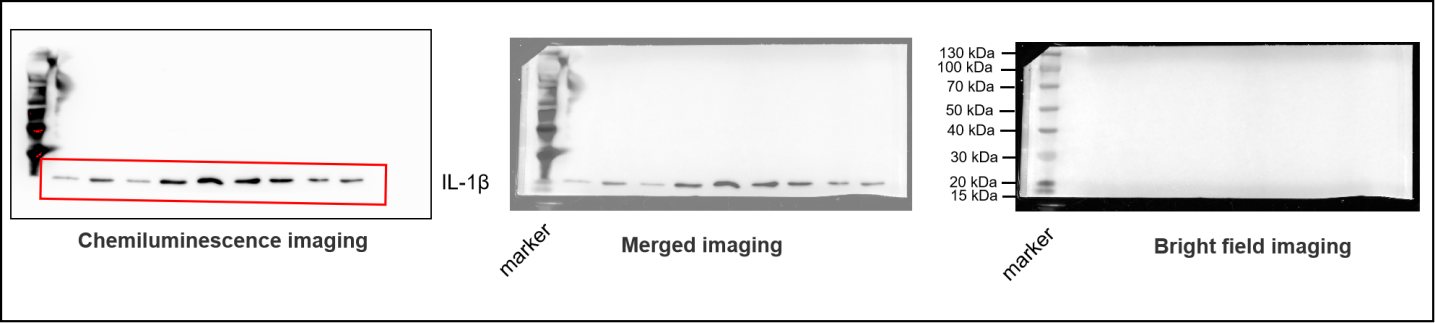


**
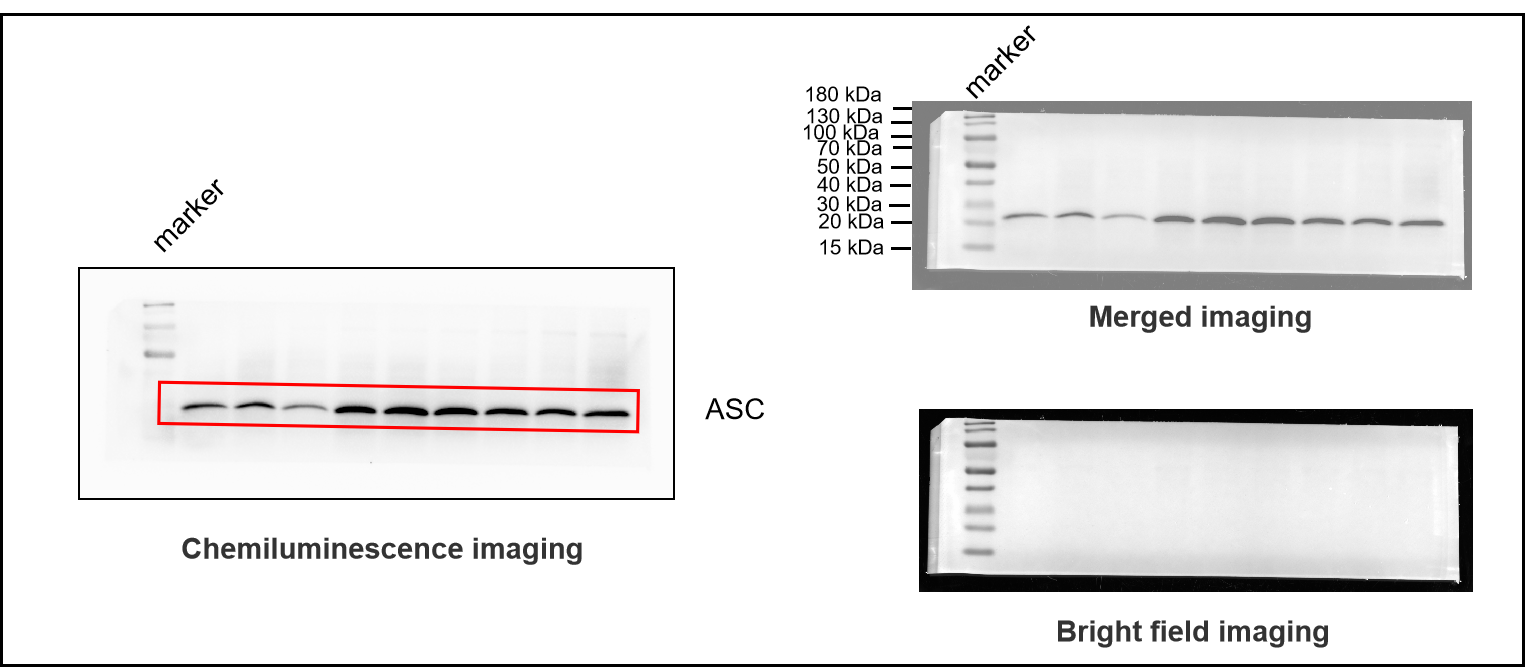

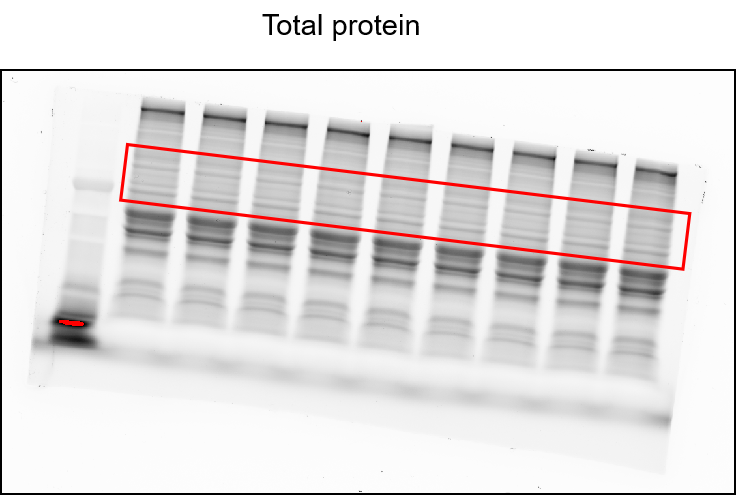

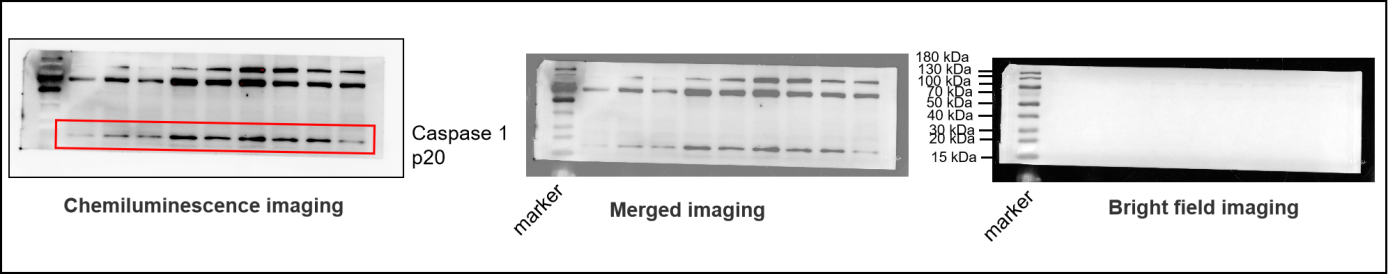

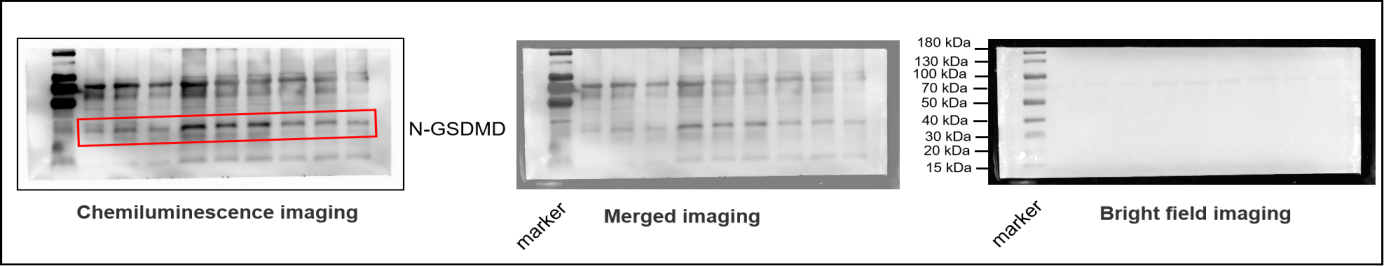

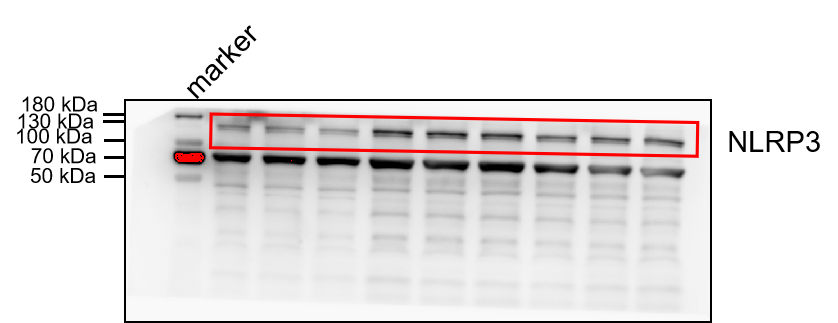
Full unedited gels/blots for Figure 7A**

**
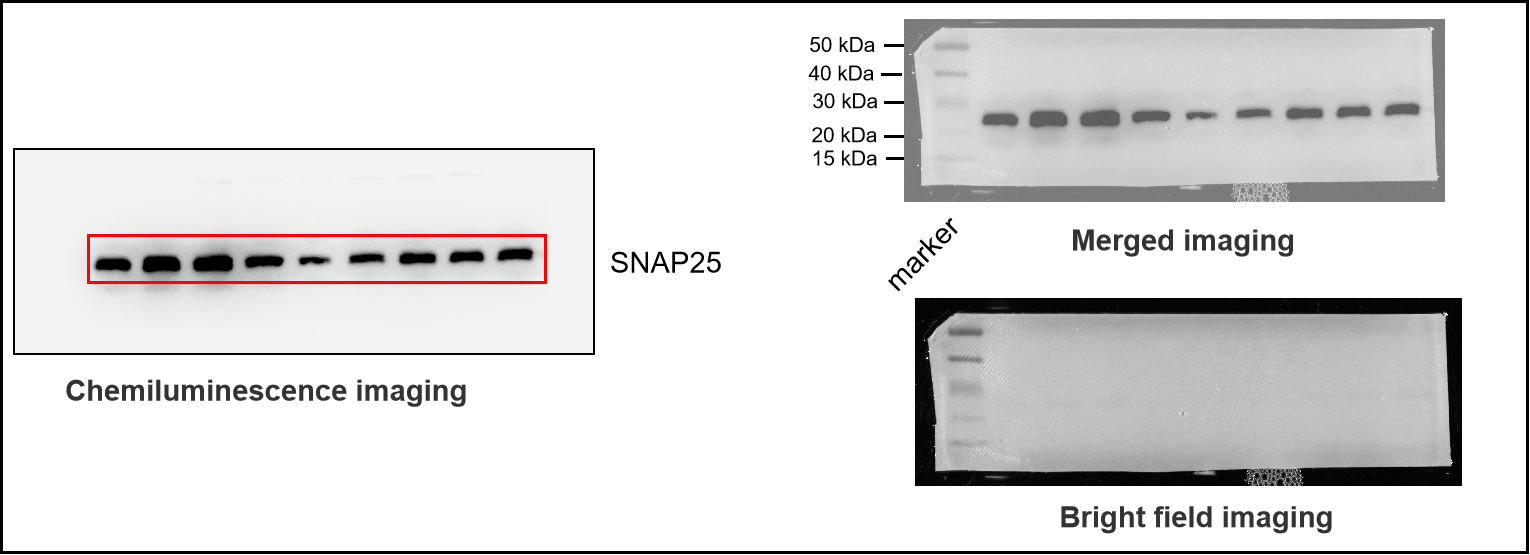

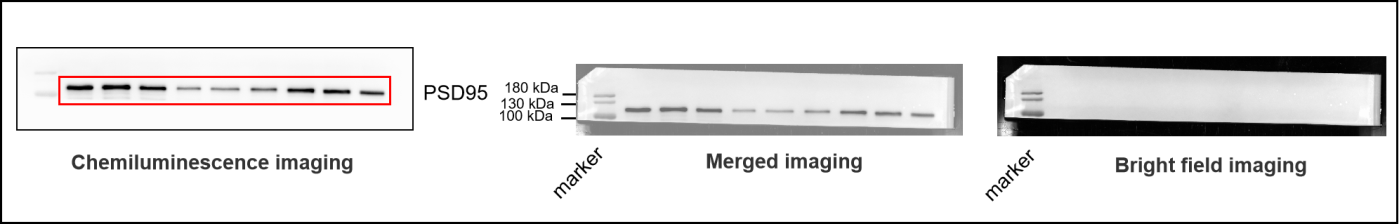
Full unedited gels/blots for Figure 8A**


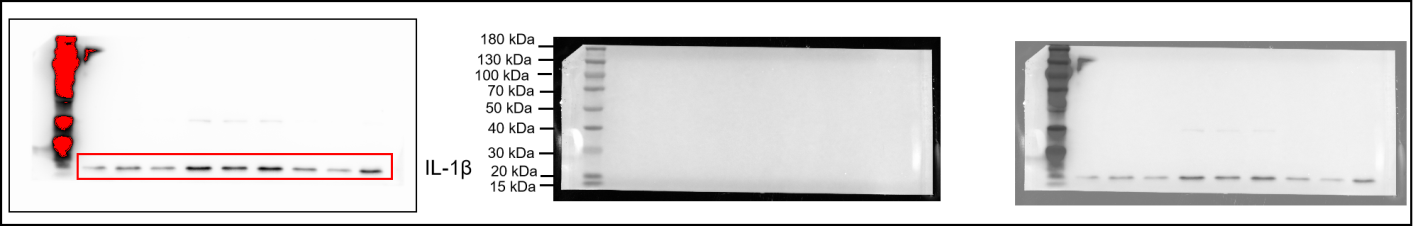

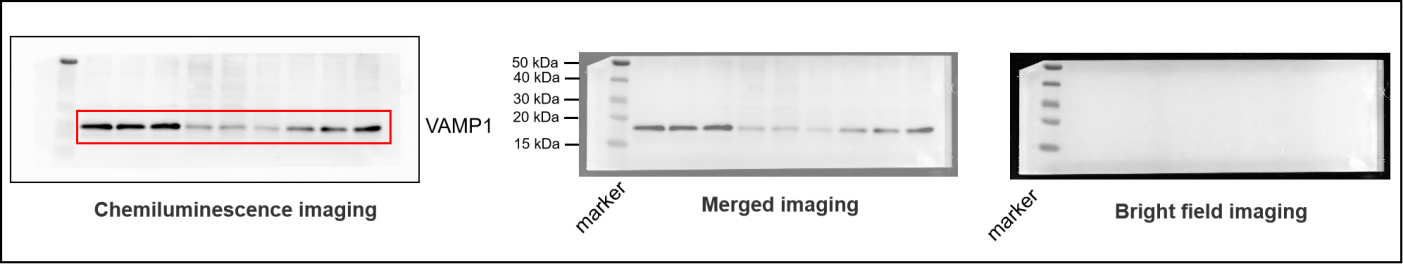

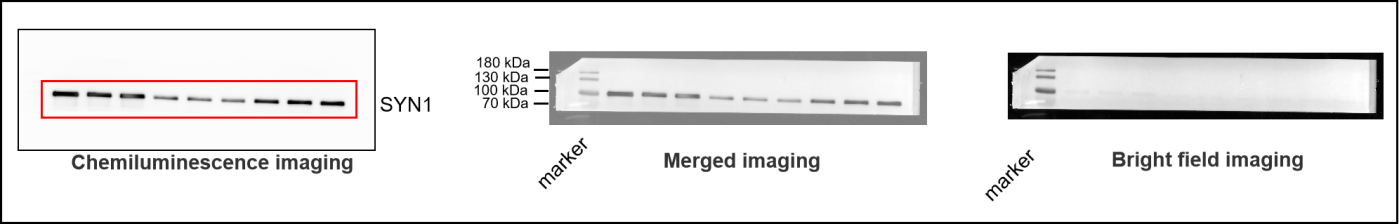


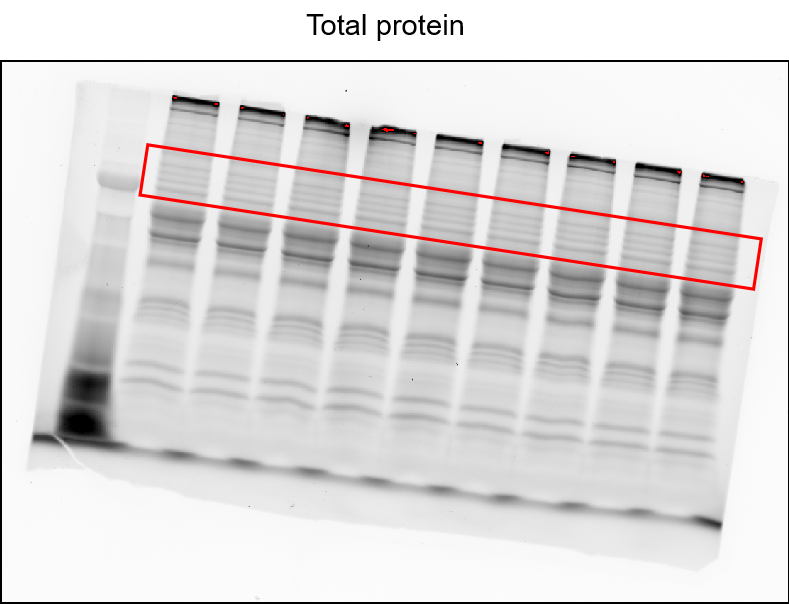


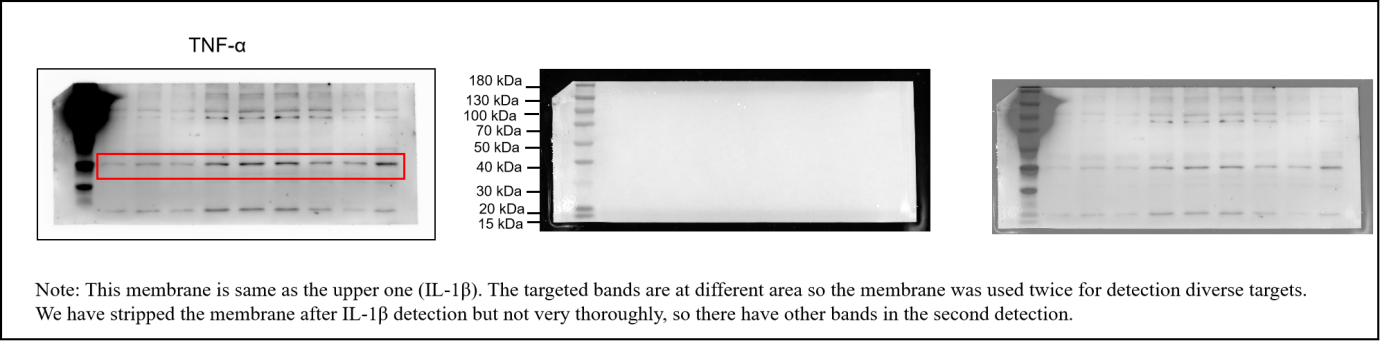

Supplement: Supplementary file 1 — Appendix S1 [file CNS-30-e14408-s001.docx]
